# Optimal 10-Aminoartemisinins With Potent Transmission-Blocking Capabilities for New Artemisinin Combination Therapies–Activities Against Blood Stage *P. falciparum* Including *Pf*KI3 C580Y Mutants and Liver Stage *P. berghei* Parasites

**DOI:** 10.3389/fchem.2019.00901

**Published:** 2020-01-10

**Authors:** Ho Ning Wong, Vivian Padín-Irizarry, Mariëtte E. van der Watt, Janette Reader, Wilna Liebenberg, Lubbe Wiesner, Peter Smith, Korina Eribez, Elizabeth A. Winzeler, Dennis E. Kyle, Lyn-Marie Birkholtz, Dina Coertzen, Richard K. Haynes

**Affiliations:** ^1^Centre of Excellence for Pharmaceutical Sciences, Faculty of Health Sciences, North-West University, Potchefstroom, South Africa; ^2^Center for Tropical & Emerging Global Diseases, Coverdell Center, University of Georgia, Athens, GA, United States; ^3^Malaria Parasite Molecular Laboratory, Department of Biochemistry, Genetics and Microbiology, Institute for Sustainable Malaria Control, University of Pretoria, Pretoria, South Africa; ^4^Division of Clinical Pharmacology, Department of Medicine, Groote Schuur Hospital, University of Cape Town, Cape Town, South Africa; ^5^School of Medicine, University of California, San Diego, La Jolla, CA, United States

**Keywords:** malaria, gametocytes, sporozoites, amino-artemisinins, transmission-blocking

## Abstract

We have demonstrated previously that amino-artemisinins including artemiside and artemisone in which an amino group replaces the oxygen-bearing substituents attached to C-10 of the current clinical artemisinin derivatives dihydroartemisinin (DHA), artemether and artesunate, display potent activities *in vitro* against the asexual blood stages of *Plasmodium falciparum* (*Pf*). In particular, the compounds are active against late blood stage *Pf* gametocytes, and are strongly synergistic in combination with the redox active drug methylene blue. In order to fortify the eventual selection of optimum amino-artemisinins for development into new triple combination therapies also active against artemisinin-resistant *Pf* mutants, we have prepared new amino-artemisinins based on the easily accessible and inexpensive DHA-piperazine. The latter was converted into alkyl- and aryl sulfonamides, ureas and amides. These derivatives were screened together with the comparator drugs DHA and the hitherto most active amino-artemisinins artemiside and artemisone against asexual and sexual blood stages of *Pf* and liver stage *P. berghei* (*Pb*) sporozoites. Several of the new amino-artemisinins bearing aryl-urea and -amide groups are potently active against both asexual, and late blood stage gametocytes (IC_50_ 0.4-1.0 nM). Although the activities are superior to those of artemiside (IC_50_ 1.5 nM) and artemisone (IC_50_ 42.4 nM), the latter are more active against the liver stage *Pb* sporozoites (IC_50_ artemisone 28 nM). In addition, early results indicate these compounds tend not to display reduced susceptibility against parasites bearing the *Pf* Kelch 13 propeller domain C580Y mutation characteristic of artemisinin-resistant *Pf*. Thus, the advent of the amino-artemisinins including artemiside and artemisone will enable the development of new combination therapies that by virtue of the amino-artemisinin component itself will possess intrinsic transmission-blocking capabilities and may be effective against artemisinin resistant falciparum malaria.

## Introduction

**Figure 1:**
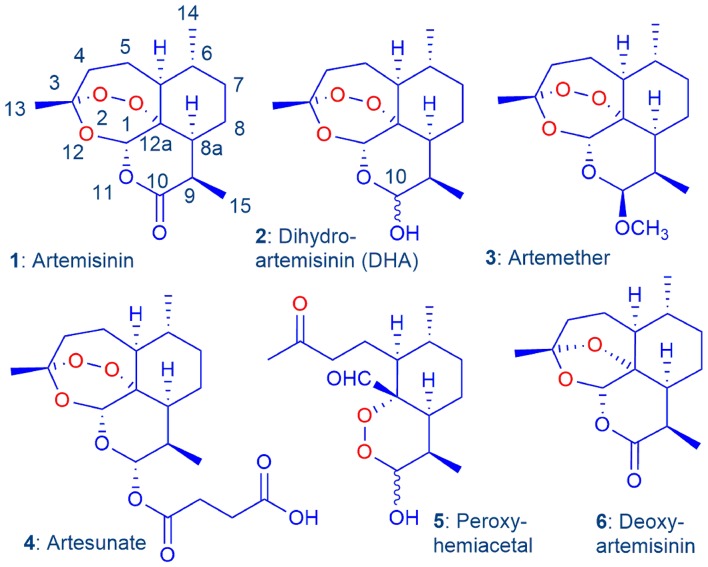
Artemisinin **1** and current clinical derivatives: dihydroartemisinin (DHA) **2**, artemether **3**, and artesunate **4**. The latter two are rapidly converted into DHA *in vivo* via metabolism or facile hydrolysis respectively. As a hemiacetal, DHA rearranges irreversibly under physiological conditions into the peroxyhemiacetal **5** that in turn rearranges to the inert deoxyartemisinin **6** (Haynes et al., 2007).

The introduction by the Chinese during the 1970s and 1980s of the antimalarial drug artemisinin **1** and its reduced derivative dihydroartemisinin (DHA) **2**, the latter which was converted into the lactol ether artemether **3**, and the hemiester artesunate **4** (Figure 1), ushered in a new era for the treatment of malaria (Brossi et al., 1988; Haynes, 2006). The introduction of the artemisinins was particularly opportune, given that the hitherto most widely-used drug chloroquine (CQ) had become essentially ineffective due to the emergence in Cambodia in the 1960s and the rapid spread of the CQ-resistant strain of the principal parasite *Plasmodium falciparum* (*Pf*) that causes malaria (Krogstad et al., 1987). Eventually, in line with the WHO recommendation, the artemisinins were combined with longer half-life antimalarial drugs such as piperaquine, mefloquine, lumefantrine, or other, in artemisinin combination therapies (ACTs). These were subsequently used with considerable success in the treatment of malaria (Adjuik et al., 2002; Cui and Su, 2009; Eastman and Fidock, 2009; Wells et al., 2009; Angus, 2014). However, increasing parasite clearance times in patients treated with ACTs in Cambodia began to be recorded after 2000 in the region where CQ resistance was first reported (Noedl et al., 2008; Amaratunga et al., 2014). More recently, increasing tolerance of the parasite to the longer half-life partner drugs in the ACT including piperaquine and mefloquine were recorded, eventually leading to overt treatment failures with ACTs (Duru et al., 2015; Leang et al., 2015; Spring et al., 2015; WHO, 2019).

The enhanced tolerance of *Pf* parasites to the artemisinins correlates with the induction of quiescence in early ring blood-stage parasites in response to drug pressure; thus parasite development is arrested, resulting in increased parasite clearance times (Dondorp et al., 2009). As a heritable trait, the increased tolerance arising via induction of ring-stage quiescence is now defined as the biomarker for artemisinin resistance (Anderson et al., 2010). The resistant phenotypes may carry point mutations, most commonly C580Y, in the *Pf* Kelch 13 propeller domain (*Pf* K13) (Ariey et al., 2014; Takala-Harrison et al., 2014; Miotto et al., 2015; Spring et al., 2015; Kheang et al., 2017; Sá et al., 2018). The transcription factor *Pf* phosphatidylinositol-3-kinase (*Pf* PI3K) in binding to *Pf* K13 mediates ubiquitinylation for protein degradation on the proteasome. The accepted thesis for induction of artemisinin resistance holds that DHA **2**, used as a clinical artemisinin in its own right, or as the principal metabolite of the clinical artemisinins artemether **3** and artesunate **4**, competitively binds to *Pf* PI3K which prevents the kinase binding to *Pf* K13, thereby inhibiting ubiquitinylation and proteasome degradation and leading to cell-cycle arrest and quiescence (Mbengue et al., 2015; Van Hook, 2015). However, the “DHA-binding” thesis is open to question (Coertzen et al., 2018). The notable lability of DHA under physiological conditions wherein DHA rearranges irreversibly via ring-opening and closure to the peroxyhemiacetal **5** (Figure 1) (Haynes et al., 2007; Jansen, 2010; Parapini et al., 2015) precludes binding of the intact molecule to *Pf* KI3. No experimental evidence for binding, as opposed to a best fit generated by *in silico* modeling of the intact DHA, was adduced in this respect. In addition, it is apparent that structurally diverse artemisinin derivatives and synthetic trioxolanes are affected to varying degrees by the resistant phenotypes (Lanteri et al., 2014; Siriwardana et al., 2016; Straimer et al., 2017). Overall, artemisinin resistance more likely reflects modulation of redox-sensitive signal transduction pathways according to other closely-related systems (Kim et al., 2005; Liu et al., 2012; Okoh et al., 2013). Thus, the inhibition equates with an enhanced response to the oxidative stress induced by the artemisinin via generation of reactive oxygen species (ROS) (Haynes et al., 2011, 2012; Coertzen et al., 2018). The origins of the stress response are in accord with one of the conceptual models for mechanism of action of artemisinins: this posits enhancement of oxidative stress brought about by the facile oxidation by artemisinins of reduced flavin cofactors of flavin disulfide reductases important for maintaining redox homeostasis in the malaria parasite (Haynes et al., 2011, 2012). More recent work indicates that resistance indeed arises through enhanced adaptive responses to oxidative stress (Bridgford et al., 2018; Rocamora et al., 2018).

Malaria mortality world-wide continues to decrease—the 405,000 deaths recorded in 2018 represent a 3% decline from 2017, and this seemingly encouraging development has a parallel in the report of a slight decrease in incidence of the disease, largely in Africa, from 231 million cases in 2017 to 228 million in 2018 (WHO, 2019). Against this, the rate of decrease of mortality and incidence has slowed since 2015, and it now appears that the WHO global technical strategy for malaria milestones for morbidity in 2025 and 2030 will not be achieved. In addition, the ongoing spread of artemisinin-resistant parasites and the decrease in the efficacies of the current ACTs are alarming, and the situation will be especially prejudiced if ingress into Africa occurs (Paloque et al., 2016; Thanh et al., 2017; Woodrow and White, 2017). In the face of the foregoing, a reappraisal of the use of current artemisinins and the partner drugs in the combinations is urgently required (Sá et al., 2018).

Whilst neurotoxicity of the clinical artemisinins, principally of DHA, is long known (Wesche et al., 1994; Nontprasert et al., 2000), this has not usually been recorded in malaria patients submitted to normal treatment regimens (Kissinger et al., 2000; Van Vugt et al., 2000; Hien et al., 2003). However, there are apparent exceptions (Toovey and Jamieson, 2004), and the perception of a neurotoxic burden is enhanced by the report of a fatality due to an artesunate overdose (Campos et al., 2008). Overall, the persistence with the widespread clinical use and development of yet newer formulations of DHA in combination therapies must be questioned, given the literature data indicative of neurotoxicity of DHA (Toovey, 2006), its reduced efficacy against artemisinin-resistant parasites (Siriwardana et al., 2016; Hamilton et al., 2019), its intrinsic instability (Haynes et al., 2007; Jansen, 2010; Parapini et al., 2015), and reports of major treatment failures, especially of the DHA-piperaquine ACT in Thailand, Cambodia, and Vietnam (WHO, 2019). Thus, artemisinins that do not undergo metabolism to DHA and are not neurotoxic have to be employed. However, the overarching aspects now are that for the drug combinations containing the new artemisinin to be applied successfully in regions of endemic artemisinin-resistant malaria, the artemisinin-resistant parasites have to be killed and transmission blocked (Alonso et al., 2011). Thus, in addition to the new artemisinin component, we need to examine with particular care the combination partners, and if these are likely to affect quiescent ring stage parasites so as to “protect” the artemisinin. We proposed use of the new artemisinin, which we term an *oxidant* drug by virtue of its ability to irreversibly oxidize reduced flavin cofactors, in combination with a *redox* (or “pro-oxidant”) drug such as a phenothiazine, e.g., methylene blue (MB), phenoxazine, naphthoquinone (Sidorov et al., 2016), quinone-imine, redox metal chelating agent (Parkinson et al., 2019), or other (Kubota and Gorton, 1999; Dharmaraja, 2017), with a third drug with a different mode of action (Coertzen et al., 2018). MB oxidizes reduced flavin cofactors of flavin disulfide reductases just as does the artemisinin (Haynes et al., 2011, 2012). However, the reduced conjugate leucomethylene blue (LMB) is rapidly reoxidized by oxygen to MB, a process that also generates ROS and initiates redox cycling of the MB (Buchholz et al., 2008). Naphthoquinones are likely to act in the same way as these oxidize reduced flavin cofactors of other flavoenzymes (Sollner and Macheroux, 2009). The benefit of combining the oxidant artemisinin with the redox drug accrues through the artemisinin abruptly inducing oxidative stress that is then maintained or enhanced by redox cycling of the MB or other redox active drug (Coertzen et al., 2018). The specific benefits bestowed by MB are potent activity against early asexual ring stage parasites (Akoachere et al., 2005) and against transmissible blood stage gametocytes (Adjalley et al., 2011). The lead benzo[α]phenoxazine SSJ-183, that is redox-active (Kubota and Gorton, 1999), like MB is active against blood stage asexual parasites, in particular ring-stage parasites (Shi et al., 2011; Schleiferböck et al., 2013). For the third drug, we consider the use of quinolones, whose antimalarial activity is well-established (Ryley and Peters, 1970; Beteck et al., 2014) and have a distinct target in blocking the quinol reductase site of the parasite mitochondrial cytochrome *bc*_1_ complex (Stickles et al., 2016). Details of our initial work on the quinolone component have been published (Beteck et al., 2018).

**Figure 2:**
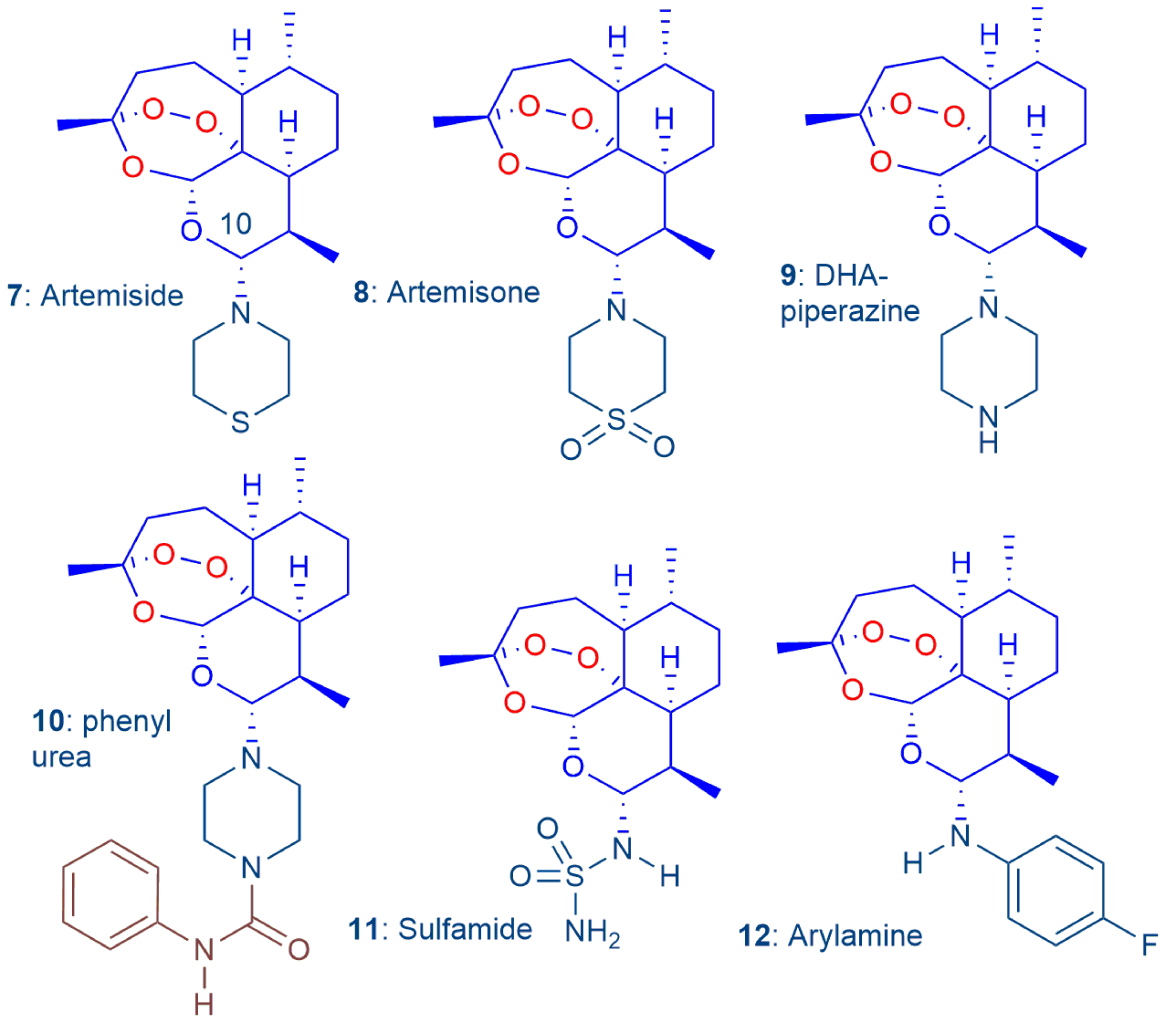
The 'basis-set' amino-artemisinins **7**–**12**, in which the exocyclic oxygen atom attached to C-10 of the clinical artemisinins (Figure 1) is replaced by a nitrogen atom, screened to select optimum derivatives (Coertzen et al., 2018).

For the artemisinin component, we commenced with artemisinins bearing amino groups at C-10 (Haynes et al., 2004), the most prominent of which are artemisone **8** and its synthetic precursor artemiside **7** (Figure 2) (Haynes et al., 2006; Chan et al., 2018). Artemisone is non-neurotoxic (Schmuck et al., 2003; Haynes et al., 2006) and is significantly more active than the current clinical artemisinins. It is not metabolized to DHA but rather to metabolites bearing unsaturation in the thiomorpholine-*S, S*-dioxide ring, and hydroxyl groups at C-5 and C-7; these also have potent antimalarial activities (Schmeer et al., 2005; Haynes et al., 2006; Nagelschmitz et al., 2008). Artemisone is safe and well-tolerated in a Phase I trial at different dose levels (Nagelschmitz et al., 2008). In a phase IIa trial with non-severe malaria patients in Thailand, artemisone was curative at one-third the dose level of the comparator drug artesunate (Krudsood et al., 2005). Artemisone is also active against cerebral malaria in a murine model. Administration at prescribed low dose levels resulted in complete cure; in contrast, at the same dose levels, each of DHA and artesunate, the latter currently used as a dual pack formulation for clinical treatment of cerebral malaria, elicited no cure (Waknine-Grinberg et al., 2010).

Thus, to guide selection of the optimum amino-artemisinins for the new combinations, we conducted a simultaneous evaluation under identical screening conditions of the efficacies of the “basis-set” amino-artemisinins artemiside **7**, artemisone **8**, the DHA-piperazine derivative **9**, its phenyl urea derivative **10**, the polar and aqueous-soluble sulfamide **11** (Haynes et al., 2006; Coertzen et al., 2018) and the readily-accessible arylamino derivative **12** (Figure 2) (Haynes et al., 2005), using as comparators DHA **2**, artemether **3**, and artesunate **4** (Coertzen et al., 2018). In particular, in view of the requirements for new antimalarial drugs to exert transmission blocking between the human host and mosquito vector, either through expunging blood-stage sexually differentiated gametocytes or in-mosquito stages of the malaria parasite (Sherrard-Smith et al., 2017), we evaluated transmission blocking capabilities of the amino-artemisinins. Additionally, our initial foray into the examination of oxidant-redox drug combinations revealed that artemiside **7** and artemisone **8** are potently synergistic with MB against both early and late stage gametocytes (Coertzen et al., 2018). Aside from vindicating our choice of an oxidant combined with a redox drug, the observation of synergism enhances the utility of the oxidant-redox drug combination for malaria treatment, and for blocking of transmission (Coertzen et al., 2018). In continuation of the programme, we seek to locate amino-artemisinins that have efficacy and toxicity properties on a par with or superior to those of artemiside **7** and artemisone **8**. Although the latter are readily obtained from DHA **2** (Haynes et al., 2006; Chan et al., 2018), we are attracted to the economically prepared DHA-piperazine **9** as a relay to inexpensive new derivatives. This is prepared by activation of DHA with oxalyl chloride-dimethyl sulfoxide (DMSO) according to our *N*-glycosylation technology followed by treatment *in situ* with piperazine in one scalable process step (Coertzen et al., 2018; Wu et al., 2018). Here we continue by converting DHA-piperazine **9** into alkyl- and arylsulfonamide derivatives, alkyl and aryl ureas, the latter bearing substitution in the aromatic ring so as to ameliorate toxicity (c.f. phenyl urea **10**, Table 1 below), and most economically, into acylated derivatives. Efficacies of the new compounds against *Pf* are assessed against asexual and gametocyte blood stage parasites. The most active compounds from the “basis set” compounds of Figure 2 and the new series are tested *in vitro* for causal prophylactic effect against *P. berghei* sporozoites expressing luciferase in a liver stage malaria model (Swann et al., 2016), and against asexual blood stages of stable phenotypes of artemisinin-resistant *Pf* carrying the C580Y mutation originally derived from Cambodian malaria patients (Hott et al., 2015).

**Table 1:** Activities *in vitro* against chloroquine-sensitive and multidrug resistant asexual blood stage *P. falciparum* and cytotoxicities of amino-artemisinins^a^.

**Compound^**a**^**	**Metabolic IC**_****50****_ **nM**			**Proliferative IC**_****50****_ **nM**^**b**^					**Cytotoxicity EC${\text{EC}}_{\text{50}}^{\text{c}}$**			
	**NF54**	**Dd2**	**RI^**d**^**	**NF54**	**K1**	**RI^**e**^**	**W2**	**RI^**f**^**	**CHO μM**	**SI^**g**^**	**HepG2 nM**	**SI^**h**^**
CQ^i^	10.0 ± 1.6	251 ± 19.9	25.1	10.0 ± 3.0	154 ± 14	15.4	233 ± 49	23.3	ND	ND	58.4	5.84
MB^i^	0.3 ± 0.8	12.6 ± 4.0	43.3	5.0 ± 0.8	6.45 ± 0.30	1.29	5.13 ± 0.31	1.03	52.6 ± 4.5	175,333	ND	ND
DHA **2**^i^	0.8 ± 0.1	5.7 ± 2.0	7.1	2.51 ± 0.19	1.51 ± 0.33	0.6	1.74 ± 0.22	0.7	25.2	10,039	ND	ND
Artemiside **7**^i^	6.0 ± 1.8	8.2 ± 1.4	1.4	1.11 ± 0.17	1.6 ± 0.4	1.47	1.75 ± 0.27	1.58	>271	>45,166	ND	ND
Artemisone **8**^i^	3.0 ± 0.8	2.7 ± 0.3	0.9	1.2 ± 0.4	1.01 ± 0.19	0.85	1.6 ± 0.4	1.36	>249	>83,000	ND	ND
DHA-piperazine **9**^i^	3.2 ± 1.4	1.7 ± 0.2	0.5	3.1 ± 0.4	1.9 ± 0.5	0.61	1.4 ± 0.7	0.45	ND	ND	ND	ND
Phenylurea **10**^i^	1.3 ± 0.8	7.5 ± 0.4	5.8	4.7 ± 1.5	2.9 ± 0.6	0.61	1.7 ± 0.5	0.36	2.4 ± 1.0	1,846	ND	ND
Sulfamide **11**^i^	10.9 ± 3.4	16.9 ± 2.6	1.55	3 ± 1	1.78 ± 0.26	0.56	2.04 ± 0.11	0.64	56.0 ± 4.6	51,376	ND	ND
Arylamine **12**^i^	7.8 ± 1.9	11.1 ± 1.0	1.41	1.3 ± 0.6	0.64 ± 0.10	0.48	3 ± 1	2.55	2.9 ± 1.4	371	ND	ND
**DHA piperazine sulfonamides**												
**13**	ND	ND	ND	2.7 ± 0.6	3.1 ± 0.1	1.1	4.4 ± 1.0	1.6	ND	ND	2,720	1007.4
**15**	1.3 ± 0.5	3.54 ± 0.32	1.8	2.7 ± 0.7	3.4 ± 0.3	1.3	5.4 ± 0.5	2.0	>177	65,555	1,953	723
**16**	1.13 ± 0.12	7.46 ± 1.07	5.7	3.4 ± 0.6	5 ± 0	1.5	4.6 ± 0.2	1.4	>178	52,352	430.9	126.7
**17**	2.0 ± 0.6	6.2 ± 0.7	3.1	3.1 ± 0.2	4.85 ± 1.1	1.6	4.6 ± 0.2	1.5	>196	6,322	637.1	205.5
**18**	15.3 ± 2.0	74.31 ± 6.42	4.9	13.9 ± 1.2	22.0 ± 2.7	1.6	23.8 ± 1.4	1.7	153.9	11,071	217.3	15.63
**19**	1.65 ± 0.04	9.8 ± 0.4	5.9	3.4 ± 0.4	4.2 ± 0.1	1.2	4.8 ± 0.5	1.4	>196	57,647	558	164.1
**DHA piperazine ureas**												
**22**	2.11 ± 0.53	24.7 ± 2.79	11.7	1.87 ± 0.12	1.12 ± 0.08	0.6	1.48 ± 0.14	0.8	2.92	1,561	615.9	329.4
**23**	1.83 ± 0.2	12.2 ± 1.9	6.7	1.55 ± 0.08	1.12 ± 0.13	0.7	1.41 ± 0.09	0.9	185	119,354	453	292.3
**24**	1.21 ± 0.04	9.2 ± 1.9	7.6	1.3 ± 0.1	0.85 ± 0.1	0.65	1.16 ± 0.17	0.9	204	156,923	339.5	36.9
**DHA piperazine amides**												
**27**	0.49 ± 0.04	13.2 ± 9.3	26.9	3.4 ± 0.4	3.5 ± 2.4	1.0	3.0 ± 0.4	0.9	150.4	44,235	135.5	39.85
**28**	1.06 ± 0.19	1.5 ± 0.6	1.4	2.8 ± 0.2	2.1 ± 1.1	0.75	2.7 ± 0.2	1.0	189.9	67,821	189.8	67.8
**DHA sulfamides**												
**29**	1.68 ± 0.18	6.4 ± 1.4	3.8	4.3 ± 0.4	4.9 ± 0.9	1.1	3.3 ± 0.7	0.8	>197	45,813	172.6	40.1
**30**	1.8 ± 0.6	9.64 ± 0.6	5.4	3.3 ± 0.3	4.3 ± 1.3	1.3	3 ± 1	0.9	34.33	10,393	311.2	94.3

a *Structures in Figures 1, 2 and Schemes 1, 2; P. falciparum NF54 CQ sensitive; P. falciparum Dd2: chloroquine (CQ), pyrimethamine resistant; K1: CQ, pyrimethamine, mefloquine, cycloguanil resistant; W2: CQ, quinine, pyrimethamine, cycloguanil resistant*.

b *Results for proliferative (SYBR Green I) assays are from three independent biological replicates, each performed as technical triplicates; ±SEM*.

c *Cytotoxicity studies using the MTT assay against CHO cells and HepG2 cells using the Cytoselect LDH cytotoxicity assay kit were performed for a single independent biological repeat, each performed as technical duplicates/triplicates, ± SD*.

d *Resistance index (RI) = IC_50_ Dd2/IC_50_ NF54*.

e *RI = IC_50_ K1/IC_50_ NF54*.

f *IC_50_ Dd2/IC_50_ NF54*.

g *Selectivity index (SI) = EC_50_ CHO/IC_50_ NF54 proliferative assay*.

h *SI = EC_50_ HepG2/IC_50_ NF54*.

i *Data from Coertzen et al. (2018)*.

The best overall compounds will be carried forward for development of new drug combinations. Accessibility and stabilities are factored into the ultimate choice: from a synthetic standpoint, the preparations must be economic, and the products must be thermally and metabolically stable relative to the current clinical artemisinins.

## Materials and Methods

### Synthetic Chemistry

The full details of the reagents, instrumentation and the procedures used to synthesize DHA-piperazine **9** and its conversion into the sulfonamides **13**–**19**, the ureas **20**–**24**, and the amides **25**–**28** (Scheme 1) are given together with characterization data for the products in the Chemistry section in Supplementary Material. Similarly, preparation and data for the substituted analogs **29** and **30** of the sulfamide **11** (Scheme 2) are also given.

**Scheme 1:**
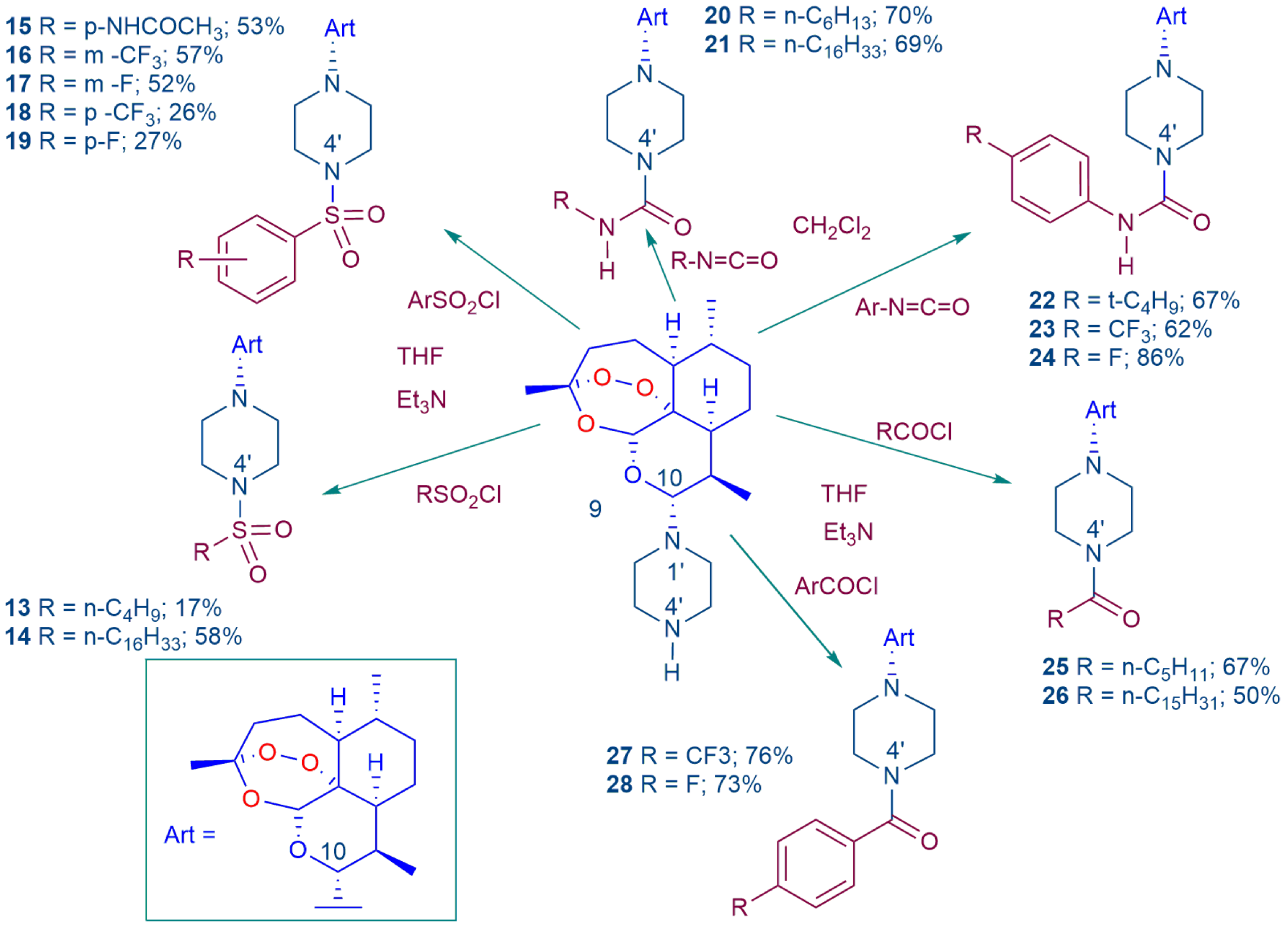
Conversion of DHA-piperazine **9** (Coertzen et al., 2018; Wu et al., 2018) into piperazine sulfonamide derivatives **13**–**19**, urea derivatives **20**–**24**, and amide derivatives **25**–**28**. The DHA-piperazine **9** was treated with the electrophile in tetrahydrofuran (THF) in the presence of triethylamine (Et_3_N) or in dichloromethane at room temperature. Yields are for products isolated by chromatography for reactions conducted on a 2.3 mmol scale with **9**.

**Scheme 2:**
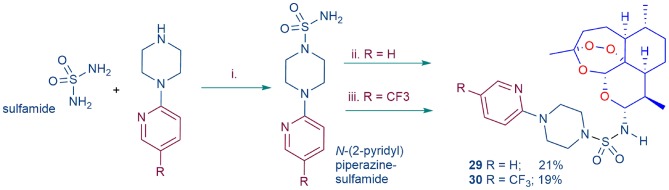
Preparation of substituted analogs of DHA-sulfamide **11** (Figure 2). i. Sulfamide (1.0 equiv.), *N*-(2-pyridyl) piperazine, or *N*-(4-trifluoromethyl-2-pyridyl) piperazine (1.0 equiv.), dimethoxyethane, reflux; ii. DHA-TMS ether, TMSBr in dichloromethane (Haynes et al., 2003, 2004), then *N*-(2-pyridyl)piperazine sulfamide (R = H); iii. DHA, COCl_2_-DMSO in toluene (Chan et al., 2018; Wu et al., 2018), then *N*-(4-trifluoromethyl-2-pyridyl)piperazine sulfamide (R = CF_3_); yields based on amount of DHA-TMS ether or DHA used.

### Biological Activities

#### *In vitro* Maintenance of Asexual Parasites and Gametocyte Production

*P. falciparum* parasites (NF54, K1, and W2) were cultured *in vitro* in human erythrocytes (A^+^ or O^+^) under 90% N_2_, 5% CO_2_, and 5% O_2_ atmospheric conditions with supplemented RPMI 1640 media (Sigma Aldrich) containing Albumax II, as previously described (Verlinden et al., 2011). This study was carried out according the guidelines set out by the Faculty of Health Sciences Ethical Committee, Ethical Clearance no. EC-120821/077. The protocol was approved by the Faculty of Health sciences ethical committee at the University of Pretoria. All subjects gave written informed consent in accordance with the Declaration of Helsinki. Parasite proliferation was monitored microscopically using Giemsa stained smears. Synchronized ring stage parasites (>95%) were obtained using a 5% D-sorbitol (Sigma Aldrich) treatment. Gametocytogenesis was induced and maintained through a combination of glucose depletion and a decrease in hematocrit from a >95% synchronized, ring stage asexual population (~10% parasitemia) as previously described (Reader et al., 2015). Gametocyte cultures were kept stationary under 90% N_2_, 5% CO_2_, and 5% O_2_ atmospheric conditions at 37°C and treated with 50 mM *N*-acetyl-glucosamine (NAG) to eliminate residual invasion of asexual parasites.

#### *In vitro* Antimalarial Assays Against Asexual *P. falciparum* Parasites

Working solutions of the derivatives were prepared from a 10 mM stock solution in 100% DMSO in supplemented RPMI 1640 media containing Albumax II with a final DMSO concentration of <0.1%, previously determined as being non-toxic to intraerythrocytic asexual parasites and gametocytes. Dose responses were assayed using a two-fold serial drug dilution on *in vitro* >95% ring stage intraerythrocytic *P. falciparum* parasites at 37°C under 90% N_2_, 5% CO_2_, and 5% O_2_ atmospheric conditions, detecting both parasite lactate dehydrogenase (pLDH) activity as a metabolic marker following a 48 h drug exposure (1.5–2% parasitemia and 2% hematocrit; Makler et al., 1993) and SYBR Green I fluorescence as proliferative marker following a 96 h drug exposure (1% parasitemia and 1% hematocrit). Activity against *P. falciparum* drug sensitive NF54 and resistant Dd2 (resistant to CQ, pyrimethamine, mefloquine, and cycloguanil), K1 (resistant to CQ, quinine, pyrimethamine, and cycloguanil), and W2 (resistant to CQ, quinine, pyrimethamine, and cycloguanil) strains were evaluated. Data analysis was performed using GraphPad Prism 7. Activity data for the compounds are averages of at least three independent biological replicates, each performed in technical triplicates; the results are expressed as the compound concentration at which 50% parasite viability/proliferation is affected (IC_50_).

#### *In vitro* Cytotoxicity Determination Against Mammalian Cells

Cytotoxicity (EC_50_) was determined against Chinese hamster ovarian (CHO) using a 3-(4,5-dimethylthiazol-2-yl)-2,5-diphenyltetrazolium bromide (MTT) assay and Caucasian hepatocellular carcinoma cells (HepG2) using the Cytoselect LDH cytotoxicity assay kit (Cell Biolabs). The assays were performed for single biological repeats, in technical triplicates (Mosmann, 1983; Rubinstein et al., 1990).

#### *In vitro P. falciparum* Gametocytocidal Assays

Gametocytocidal activity was determined using the luciferase reporter line (Reader et al., 2015) to derive dose responses with two-fold serial drug dilutions for 48 h against early stage gametocytes (day 5 post-induction population, >90% stage I–III) and 10-fold serial drug dilutions for 72 h against late stage gametocytes (day 10 post-induction population, >90% late stage IV–V) (2% gametocytemia, 2% hematocrit), at 37°C under 90% N_2_, 5% CO_2_, and 5% O_2_ atmospheric conditions. Data are the averages per compound are from at least three independent biological replicates, unless otherwise indicated, each performed in technical triplicates, and results expressed as the compound concentration at which 50% parasite viability is affected (IC_50_).

#### Stage-Specificity and Kill Kinetic Evaluation of Compounds Against Late Stage Gametocytes

Late stage gametocytes, stage III, IV/V (10% stage III, 50% stage IV, 40% stage V population on day 10 post-induction) or mature stage V (>95% stage V on day 13 post-induction) were used to determine the differential stage specificity and kill kinetics (speed-of-action) of the compounds. Dose responses were determined using the luciferase reporter line, exposed to 10-fold serial drug dilutions for 72 h at 37°C under 90% N_2_, 5% CO_2_, and 5% O_2_ atmospheric conditions. Treatment for shorter periods (e.g., 24 h) did not result in accurate dose response determination of any compound, irrespective of gametocyte population used, confirming the insensitivity of gametocytes to short periods of perturbation (Adjalley et al., 2011). Additionally, a drug washout step was included by replacing the drug-containing spent medium with fresh media (without drug) followed by a further 24 h incubation prior to measuring luciferase activity. Gametocyte viability was monitored morphologically and by detection of exflagellation events at the initiation and completion of the experiment with a 20 min exposure to 1 mM xanthurenic acid at room temperature. Population compositions were determined microscopically using Giemsa stained smears before (0 h) and after (72 h/72 + 24 h) incubations for both treated and untreated populations at 2 × IC_50_. Data are the average from a single independent biological experiment with technical triplicates, error bars indicate ±SD.

#### *In vitro* Assays Against *P. berghei* Liver Stage Development (Causal Prophylactic Mode)

Potential causal prophylactic activity was tested as previously described (Antonova-Koch et al., 2018). Briefly, HepG2-CD81 cells were seeded into 1,536 well plates containing 50 nL of test and control compounds diluted into DMSO. Approximately 24 h later, ~1,000 *P. berghei* sporozoites (*P. berghei* ANKA GFP-Luc-SM_con_) in screening media were added to each well. Cells were incubated for 48 h at 37°C. Next, 2 μL of luciferin reagent (Promega BrightGlo) was added to each well and luciferase activity was detected using a Perkin Elmer Envision plate reader. IC_50_ values were determined in CDD vault (https://www.collaborativedrug.com/) normalized to maximum and minimum inhibition levels for the positive (atovaquone, 0.25 μM) and negative (DMSO) control wells. In parallel, plates were processed without sporozoites for HepG2 toxicity measurements. Here Promega CellTiterGlo (2 μL) was added instead of BrightGlo with curve fitting as above using puromycin (25 μM) as a positive control and (DMSO) as negative control wells.

#### *In vitro* Antimalarial Assays Against Asexual Artemisinin Resistant Cambodian Field Isolates

Two artemisinin resistant field strains, containing the C580Y point mutations were cloned into stable resistant phenotypes ARC08-22 (4G) and PL08-09 (5C) using the *in vitro* method as previously described (Hott et al., 2015). The time-zero (T_0_) [^3^H]-hypoxanthine drug susceptibility assay was performed to determine the susceptibility of the compounds (**Table 4**) against the artemisinin resistant clones. Dihydroartemisinin **2** was included as a control due its lower activity against the resistant clones. Briefly, [^3^H]-hypoxanthine and the drug of interest was added to *in vitro* ring stage cultures at the beginning of the assay (T_0_) and incubated for 48 h at 37°C, followed by harvesting of radiolabelled cells (T_48_) to determine the respective IC_50_ and IC_90_ values.

## Results

### Chemistry

DHA-piperazine **9** was treated with alkyl and aryl sulfonyl halides to provide the sulfonamides **13**–**19**, alkyl and aryl isocyanates to provide the ureas **20**–**24**, and with acyl halides the amides **25**–**28** (Scheme 1). Whilst reactions of the aryl electrophiles proceeded well, the alkyl counterparts were less successful. For preparation of the alkyl sulfonamides, medium chain length alkanesulfonyl chlorides, e.g., *n*-decanesulfonyl chloride did not react consistently, and for the alkyl ureas, use of *tert*-butyl or 1-adamantyl isocyanate unfortunately did not give discrete products. Previously, the 4′-*N*-methanesulfonyl piperazine derivative was prepared by coupling of *N*-methanesulfonylpiperazine with DHA according to our original method for preparing amino-artemisinins (Haynes et al., 2004, 2006). Thus, the method of commencing with DHA-piperazine **9** offers an attractive flexibility in providing new derivatives carrying diverse substitution at N-4′. The methods complement others wherein glycosides (Wu et al., 2018) and ligands incorporating cholesterol (Morake et al., 2018) may readily be attached to N-4′ of DHA-piperazine.

Whilst among the basis-set artemisinins (Figure 2), the DHA-sulfamide derivative **11** elicits good activities against asexual blood stage parasites, it is less active against gametocytes (Table 1). Nevertheless, the sulfamide scaffold is attractive because it is thermally and hydrolytically stable, and confers water solubility (DHA-sulfamide **11** 294 mg/L; c.f. artemisone **8** 89 mg/L) (Haynes et al., 2006). Thus, the more polar substituted piperazine sulfamide analogs were prepared for comparative purposes (Scheme 2). The *N*-substituted piperazine sulfamides, readily obtained from sulfamide and the corresponding *N*-(2-pyridyl) piperazine, were coupled with DHA either according to our original protocol involving conversion of DHA α-trimethylsilyl (TMS) ether into the β-bromide by treatment with trimethylsilyl bromide (TMSBr) (Haynes et al., 2003, 2004) or via direct conversion of DHA with oxalyl chloride-DMSO into the β-chloride (Chan et al., 2018; Wu et al., 2018), and then by treatment of the halides *in situ* with the respective *N*-(2-pyridyl) sulfamides (Scheme 2). However, yields of products **29** and **30** obtained by the respective procedures were too low to be practical, although the activity data presented below is useful from a structure-activity viewpoint.

All compounds are isomerically pure with stereochemistries as depicted. The sulfonamides **15**–**17**, ureas **22**–**24**, amides **27** and **28**, and sulfamides **29** and **30** are crystalline, and as assessed by differential scanning calorimetry (DSC) are thermally relatively stable with melting points ranging from 136 to 160°C (c.f. artemiside 161, artemisone 160°C). Full details are given in the Supplementary Information. As shall be described elsewhere for selected examples, the compounds, like artemisone **8**, are not metabolized to DHA.

### Biological Activities

#### Artemisinin-Sensitive Asexual Blood Stage Parasites

The *in vitro* activities of the new derivatives were determined against drug sensitive NF54 and multi-drug resistant Dd2, K1, and W2 intraerythrocytic asexual *P. falciparum* malaria parasite strains using both a metabolic (parasite LDH) and a proliferative (SYBR Green I fluorescence) assay readout (Table 1). These two different assay platforms provide adequate coverage of the overall activities of the compounds toward the asexual stages. With the notable exception of the long chain alkane sulfonamide **14**, alkyl ureas **20** and **21**, and amide **26** that were considerably less active (IC_50_ > 50–100 nM, data not shown), the majority of new compounds inhibited asexual *P. falciparum* metabolic activity and proliferation in the low nM range, with IC_50_ values <5 nM (Table 1). The aryl ureas **23** and **24** and the *p*-fluorophenyl amide derivative **28** showed the highest potency across both assay platforms, with IC_50_ values of ~1–2 nM for drug sensitive NF54 parasites. Somewhat unexpectedly, the aryl sulfonamide **18** showed the least activity against asexual stages, with IC_50_ values ranging from 15 to 74 nM for drug-sensitive and -resistant parasites. In general, the new compounds did not show any cross-resistance between the drug-sensitive and resistant parasites, with resistant index (RI) values <10. This contrasts with the case of CQ which displays an RI > 15 between sensitive (NF54) and CQ-resistant (Dd2, K1, W2) strains (Table 1). Gratifyingly, it is noted that whereas the phenyl urea **10** is relatively cytotoxic toward CHO cells (EC_50_ 2.4 μM, Table 1), the aryl analogs **23** and **24** bearing electron-withdrawing groups (*p*-CF_3_, -F) on the aromatic ring are appreciably less toxic (EC_50_ 185 and 204 μM respectively; Table 1).

#### Blood-Stage Gametocytes

For assessment of transmission-blocking capabilities, all compounds were screened against gametocytes using the NF54 luciferase reporter cell lines and luciferase viability assay applied previously to the basis-set artemisinins (Table 1; Coertzen et al., 2018). The new 10-amino-artemisinins showed low nM activities against both early (>90% stages I–III) and late stage gametocytes (>90% stages IV and V; Table 2), with activity levels similar to those observed against the asexual stages (Table 1). Of the derivatives, the aryl sulfonamide **15** was the most active against early stage gametocytes (IC_50_ 8 ± 2.3 nM), and together with the butanesulfonamide **13**, aryl sulfonamides **16** and **17**, and aryl amide **28** displayed sub-nanomolar IC_50_ values (0.4–0.7 nM) against late stage gametocytes. The DHA-sulfamide analog **29** was the most active of all compounds against late stage gametocytes (IC_50_ 0.04 nM), although its relative inaccessibility tempers this discovery. As with the asexual stages, the aryl sulfonamide **18** showed the least activity against early stage gametocytes, with a marginal loss of activity against late stage gametocytes as compared to the other sulfonamides. With the exception of the *p*-trifluoromethylaryl urea **23** (late stage IC_50_ 1.0 nM), the aryl ureas **22** and **24** although very active against asexual stage parasites (Table 1), were relatively less active against early and late stage gametocytes. Overall, most of the amino-artemisinins tested here were significantly more active against late stage gametocytes (*P* = 0.0006) compared to the early stage gametocytes and were equipotent against asexual parasites (*P* = 0.8880). This is apparent in the >2–578-fold preference for late stage gametocytes indicated in Table 2. This pronounced preference for late stage gametocytes is noteworthy, as most antimalarial compounds tend to show greater activity against the early stages with a loss in activity against late stage gametocytes (Peatey et al., 2012; Duffy and Avery, 2013). Thus the sub-nanomolar activities (IC_50_ 0.4–0.7 nM) of the sulfonamides **13**–**17** and **19**, and the aryl amide **28** against late stage gametocytes, as compared to the micromolar activities of most other compound classes (Delves et al., 2018) are striking.

**Table 2:** Activities of amino-artemisinins *in vitro* against early and late blood stage *P. falciparum* NF54 gametocytes^a^.

**Compound^**a**^**	**Early Stage (EG, Luc 48 h) IC_**50**_ nM^**b**^**	**Late Stage (LG, Luc 72 h) IC_**50**_ nM^**c**^**	**Fold change preference ratio: EG to LG**	**Fold change preference ratio: LG to EG**
MB^d^	95.0 ± 11.3	143.0 ± 16.7	1.5	0.7
DHA **2**^d^	43.0 ± 3.9	33.66 ± 1.98	0.78	1.3
Artemiside **7**^d^	16.4 ± 1.0	1.5 ± 0.5	0.09	10.9
Artemisone **8**^d^	1.94 ± 0.11	42.4 ± 3.3	21.9	0.05
DHA-piperazine **9**^d^	54.91 ± 5.11	25.7 ± 17.5	0.47	2.1
Phenylurea **10**^d^	83 ± 2	1.70 ± 0.99	0.02	48.8
Sulfamide **11**^d^	15.0 ± 2.0	419.4 ± 59.5	28.0	0.04
Arylamine **12**^d^	38.2 ± 9.0	16.42 ± 6.38	0.4	2.3
**DHA piperazine sulfonamides**				
**13**	21.5 ± 10.4	0.5 ± 0.3	0.02	40.55
**15**	8.0 ± 2.3	0.9 ± 0.5	0.11	8.78
**16**	19.7 ± 9.9	0.4 ± 0.3	0.02	46.26
**17**	13.1 ± 7.2	0.6 ± 0.4	0.05	20.98
**18**	89.7 ± 28.8	4.8 ± 4.2	0.05	18.60
**19**	12.1 ± 4.5	0.7 ± 0.5	0.06	16.28
**DHA piperazine ureas**				
**22**	45.9 ± 9.9	17.7 ± 15.1	0.39	2.12
**23**	20.0 ± 9.4	1.0 ± 0.3	0.05	19.48
**24**	36.0 ± 10.2	17.0 ± 7.0	0.47	2.59
**DHA piperazine amides**				
**27**	43.7 ± 5.1	2.8 ± 1.8	0.06	33.13
**28**	19.5 ± 8.5	0.6 ± 0.3	0.03	15.69
**DHA sulfamides**				
**29**	24.7 ± 4.9	0.04 ± 0.03	0.00	578.76
**30**	19.3 ± 6.2	4.0 ± 3.8	0.21	4.77

a *Structures in Figures 1, 2 and Schemes 1, 2; IC_50_ values against*.

b *Early stage (>90% stage I–III)*.

c *Late stage (>90% stage IV and V) gametocytes determined using the luciferase based assay against the Luc reporter cell line. Results are representative of the mean of three independent biological replicates, performed as technical triplicates, ±SEM. Data are from a 72-h incubation period*.

d *Data from Coertzen et al. (2018)*.

**Figure 3:**
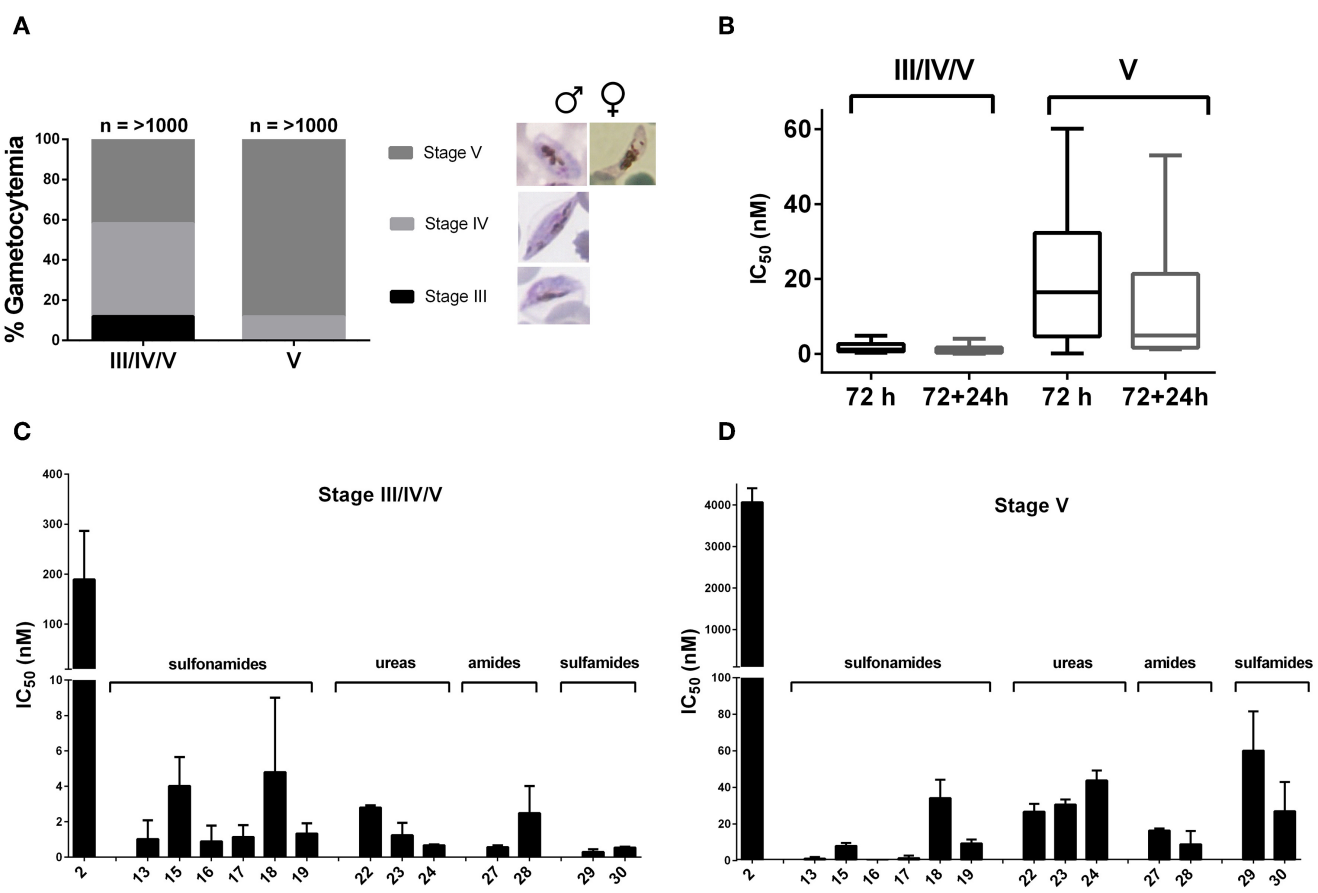
Stage-specificity and transmission blocking potential of amino-artemisinins against stage IV/V and stage V gametocytes. **(A)** Population compositions at time of treatment (0 h) for both stage III/IV/V (10% stage III, 50% stage IV, 40% stage V) or mature stage V (>95% stage V) gametocytes were determined microscopically using Giemsa stained smears (*n* ≥ 1,000 infected erythrocytes were counted). **(B)** Box-plot of mean IC_50_ values of amino-artemisinins after 72 h and 72 + 24 h treatment for both stage III/IV/V and V gametocytes. Dose-responses of DHA **2** and amino-artemisinins against a **(C)** stage III/IV/V and **(D)** stage V population using the luciferase reporter line exposed to 10-fold serial dilutions for 72 h at 37°C under 90% N_2_, 5% CO_2_, and 5% O_2_ atmospheric conditions. Data are averages of for a single independent biological experiment, performed in technical triplicates, error bars indicate ± SD.

To validate the potential transmission blocking activity of the derivatives through targeting mature stage V gametocytes, comparative kinetic studies were carried out on two separate late stage gametocyte populations. The first population consisted of ~10% stage III, 50% stage IV, and 40% stage V and the second an enriched late stage population of >95% stage V gametocytes (Figure 3A). The IC_50_ values were determined after a 72 h treatment time, including a drug washout step followed by an additional 24 h incubation to establish persistence of drug effect after washout. The amino-artemisinins showed a >100-fold loss in activity against stage V (mature stage) gametocytes compared to stage III/IV/V (immature late stage) gametocytes, even following a further 24 h incubation after drug washout (Figure 3B). This correlates with previous reports a of loss in compound activity against mature gametocyte stages (Duffy and Avery, 2013; Plouffe et al., 2016). However, *a* > 100-fold increase in activity compared to DHA **2** was observed for the amino-artemisinins against both gametocyte populations (Table 3, Figures 3C,D). Therefore, the amino-artemisinins show potent transmission blocking activity with IC_50_ values <100 nM. Particularly, the sulfonamides **13**–**17** and the amide **28** were the most potent (IC_50_ values <10 nM) against stage V gametocytes (Figure 3D).

**Table 3:** *In vitro* activities of selected amino-artemisinins against liver stage *P. berghei* and cytotoxicities^a^.

**Compound**	***P. berghei*** **sporozoites**		**Cytotoxicity EC**_****50****_	
	**IC_**50**_ nM**	**Maximum inhibition % (Conc. μM)**	**HepG2 μM**	**SI^b^**
Atovaquone	2.515 ± 0.997	94.85 ± 2.76 (0.5)	>0.25	>100
Puromycin	22.7 ± 4.525	110 ± 4.24 (5)	0.117	5.15
Artemether **3**	>10^4^	49.4 (10)	ND	ND
Artemiside **7**	81.3 ± 9.616	99.05 ± 1.34 (5)	>25.0	>308
Artemisone **8**	28.3 ± 01.273	93.35 ± 1.76 (10)	>50.0	>1767
**DHA piperazine ureas**				
**23**	82.55 ± 4.172	104.75 ± 7.42 (10)	5.45	66.0
**24**	105.5 ± 6.363	94.85 ± 2.76 (10)	5.16	48.9
**DHA piperazine amides**				
**27**	168.0 ± 55.154	108 ± 4.24 (10)	7.015	41.8
**28**	114.0 ± 15.556	104 ± 4.24 (10)	8.32	73.0

a *Structures in Figures 1, 2 and Schemes 1, 2; luciferase-expressing P. berghei ANKA GFP-Luc-SM_con_ sporozoites were allowed to invade HepG2 cells and luciferase activity was measured after 48 h; data ± SD from biological duplicate and technical quadruplicate measurements*.

b *SI = EC_50_ HepG2/IC_50_ P. berghei sporozoites. Assay was performed as previously described (Swann et al., 2016)*.

#### Liver Stage *P. berghei* Sporozoites

In continuation of the transmission blocking theme, it is important to establish activities against liver stage parasites. Selected compounds were tested *in vitro* for causal prophylactic effect against *P. berghei* sporozoites expressing luciferase in a liver stage malaria model (Swann et al., 2016). This assay uses ultra-high-throughput screening methods optimized for a 1536-well format and a HepG2 human hepatoma cell line. A cytotoxicity assay was also performed in which ATP was quantified to measure HepG2 cell viability (Table 3). Notably of the amino-artemisinins including the aryl ureas **23** and **24**, and the aryl amides **27** and **28** screened, artemisone **8** was the most active. Dose response analyses of artemisone **8** in 12-point titrations starting from 10 μM, and 2.5 μM showed that this maintained >75% inhibition of liver stage parasites down to 3 μM. To establish selectivity of the compounds for the parasite in the liver stage of infection, cytotoxicity was determined by measuring bioluminescence from the reaction between free ATP of compound-treated, uninfected hepatocytes and a luciferase reporter system. Artemiside **7** and artemisone **8** displayed no measurable IC_50_ values at 25 and 50 μM, respectively (Table 3). Thus, notably, these more established amino-artemisinins are the most highly selective against liver stage *P. berghei*.

#### Artemisinin-Resistant Asexual Blood Stage Parasites

Preliminary drug susceptibility assays performed on asexual blood stages of two artemisinin-resistant *P. falciparum* clones ARC08-22 (4G) and PL08-009 (5C) from Cambodia carrying the C580Y mutation using *Pf* W2 as a comparative artemisinin sensitive strain according to the previously recorded method (Hott et al., 2015) showed no indication of reduced susceptibility (resistance) toward the artemiside **7** and artemisone **8** (Table 4). For the new aminoartemisinins, whilst the *p*-fluorophenyl amide derivative **28** shows on average the highest potency against asexual blood stage parasites of drug-sensitive parasites (Table 1), both this compound and the *p*-trifluoromethyl analog **27** showed no indication of reduced susceptibility toward the ARC08-22 (4G) clone. Surprisingly however, the PL08-009 (5C) showed a > 20-fold decrease in susceptibility toward these two derivatives, and indeed toward the aryl urea **10**. This appears anomalous, as although these two strains have the same genotypes, the only difference is the length of their independent life cycles: the ARC08-22 (4G) clone has a 48 h life-cycle in comparison to the PL08-009 (5C) strain, which has 36 h life-cycle. It should be noted that efficacy data recorded previously on *Pf* wild-type isolates from Cambodia (CamWT) and Cam3.II (carrying the R539T *Pf* K13 mutation) engineered to produce the transfected strains CamWTC580Y (C580Y K13 mutation), Cam3.IIC580Y (C580Y K13 mutation), and Cam3.IIRev (wild-type K13 sequence) indicate that artemisone essentially retains baseline activities across all strains, as reflected in a mean IC_50_ value of 2.4 nM; in comparison the corresponding activity against W2 was reported to be 1.9 nM. For DHA, activities expressed in terms of mean IC_50_ values were 11.2 and 5.2 nM, respectively (Lanteri et al., 2014).

**Table 4:** Activity of amino-artemisinins against *P. falciparum* asexual blood stage artemisinin-resistant clones carrying the *Pf* KI3 C580Y mutation as determined with the T_0_ [^3^H]-hypoxanthine drug susceptibility assay.

**Compound^**a**^**	**W2**		**ARC08-22 (4G) (48 h lc)**^**b**^			**PL08-009 (5C) (36 h lc)**^**b**^		
	**IC_**50**_ nM**	**IC_**90**_ nM**	**IC_**50**_ nM**	**IC_**90**_ nM**	**RI^**c**^**	**IC_**50**_ nM**	**IC_**90**_ nM**	**RI^**d**^**
DHA **2**	4.58 ± 2.54	10.30 ± 5.76	6.68 ± 0.61	15.40 ± 1.39	1.5	6.41 ± 1.54	10.73 ± 3.81	1.4
Artemiside **7**	2.21 ± 0.42	4.28 ± 0.25	2.43 ± 0.13	4.85 ± 0.09	1.1	0.29 ± 0.03	0.43 ± 0.03	0.1
Artemisone **8**	1.69 ± 0.36	3.42 ± 0.45	1.62 ± 0.19	3.38 ± 0.28	1.0	0.27 ± 0.05	0.43 ± 0.07	0.2
Urea **10**	0.12 ± 0.03	0.20 ± 0.03	0.19 ± 0.02	0.36 ± 0.03	1.6	4.71 ± 0.60	6.45 ± 1.81	39.3
Sulfamide **11**	4.87 ± 0.59	7.53 ± 0.23	4.76 ± 0.25	7.55 ± 0.26	1.0	1.81 ± 0.20	2.38 ± 0.06	0.4
Arylamine **12**	4.76 ± 0.38	8.51 ± 0.34	5.51 ± 0.59	10.92 ± 0.95	1.2	15.73 ± 0.72	22.57 ± 1.00	3.3
**DHA piperazine amides**								
**27**	0.21 ± 0.04	0.41 ± 0.08	0.39 ± 0.02	0.70 ± 0.05	1.9	9.49 ± 1.05	14.06 ± 1.27	45.2
**28**	0.10 ± 0.03	0.19 ± 0.02	0.16 ± 0.01	0.43 ± 0.02	1.6	4.22 ± 0.42	5.83 ± 1.37	42.2

a *Structures in Figures 1, 2 and Scheme 1*.

b *lc = life cycle (h)*.

c *RI = IC_50_ for ARC08-22(4G)/IC_50_ for W2*.

d *RI = IC_50_ for PL08-09 (5C)/ IC_50_ for W2. Results are the mean of three independent biological replicates, performed as technical triplicates, ±SEM*.

## Discussion

Due to the emergence of resistance both toward the artemisinin component and the other traditional antimalarial drug component of the current ACTs, novel triple drug combinations need to be developed as a matter of urgency, not only in terms of their ability in expunging artemisinin-resistant blood stage parasites, but also in blocking transmission of the resistant parasites by targeting blood stage gametocytes and liver-stage sporozoites. As artemisinins induce rapid reduction of the parasitemia associated with malaria pathogenesis, it is considered the amino-artemisinins that are optimally efficacious against blood stage parasites, are non-neurotoxic, and display improved pharmacokinetic and drug metabolism profiles, including especially lack of metabolism to DHA, are best used as part of any new drug combination. Further, in order to inhibit transmission of the resistant parasites via uptake of sexually-differentiated late blood stage gametocytes from an infected human to the mosquito, drugs that kill these transmissible stages are required. Therefore, one or more drugs in the new combination should have transmission-blocking capabilities. For the artemisinin-resistant parasites, whilst ideally, the new artemisinin component should not be overtly affected by the K13 resistance phenotype, it remains to be established how effective the amino-artemisinins are in this regard (see below). Thus, the new artemisinin should be protected by the other components of the combination, namely the redox-active drug, and the third combination partner. For the last, this must have a mechanism of action distinct to those of the oxidant artemisinin and redox active component. Selected redox drugs and third combination partners are being evaluated in the same assays, as it critical to select partner drugs that may protect the artemisinin in the putative triple combination for treatment of artemisinin-resistant malaria. As noted above, we have already commenced this work by developing quinolones based on the old coccidiostat decoquinate (Beteck et al., 2018).

As our first step in achieving this aim, the new amino-artemisinins based on the DHA-piperazine **9** and the DHA-sulfamide **11** scaffolds were prepared and screened together with the artemiside **7** and artemisone **8** against drug susceptible and resistant asexual blood stage *Pf* parasites, against mature *Pf* gametocytes and against *P. berghei* liver stage sporozoites. Overall, the new aryl amino-artemisinin derivatives were active against asexual parasites, and early and late stage gametocytes and displayed good selectivity indices with respect to drug-sensitive and resistant strains, which are essentially identical with those of artemiside **7** and artemisone **8** (Table 1). The last compounds also have good selectivity indices against CHO cells; a comparison of their toxicities indicate these are less toxic than the aryl ureas **23** and **24** (Table 1). It is noteworthy that of the aryl amino-artemisinin derivatives, the *p*-trifluoromethylaryl urea **23** and *p*-fluoroaryl urea **24**, and the *p*-fluorophenyl amide **28** consistently returned outstanding activities. In particular, the urea **23** (IC_50_ 1.0 nM) and the amide **28** (IC_50_ 0.6 nM) were selectively active against late and mature stage gametocytes (Table 2, Figure 3), and in this respect have activities superior to those of artemiside **7** (IC_50_ 1.5 nM) and artemisone **8** (IC_50_ 42.4 nM) (Table 2). It has to be noted that this level of activity against transmissible blood stage parasites contrasts with the relatively poor activities of DHA **2**, artemether **3** and artesunate **4** (Adjalley et al., 2011; Plouffe et al., 2016) as seen in Figure 3. In contrast to the clinically-used artemether **3** (Table 3), artemiside **7** and artemisone **8**, and compounds **23**, **24**, and **28** are potently active against liver stage *P. berghei* sporozoites. Thus the amino-artemisinins have the potential to display prophylactic activity that is not apparent with the current clinical artemisinins (Meister et al., 2011). With respect to the artemisinin-resistant phenotypes, the reduced susceptibility observed for compounds **27** and **28** against the *Pf* KI3 C580Y PL08-09 strain (Table 4) has to be noted and queried; further screening is required to pinpoint the cause of this reduced susceptibility. In contrast, artemisone **7** and artemiside **8** are not affected, and as noted above, artemisone **8** showed no reduction in susceptibility to the laboratory-adapted K13 mutants (Siriwardana et al., 2016). Although the observation of no appreciable loss in susceptibility of these amino-artemisinins against artemisinin-resistant parasites is encouraging, further work is required in order to establish overall susceptibility of the resistant phenotypes (Witkowski et al., 2013; Kite et al., 2016; Sá et al., 2018). To this end, we are currently evaluating the effects of the amino-artemisinins on induction of quiescence in ring stage assays involving the resistant phenotypes.

The observations recorded here in affirming, and adding to, those recorded previously (Coertzen et al., 2018) indicate that the amino-artemisinins including artemiside **7** and artemisone **8** are highly active against asexual blood stage parasites, gametocytes, and sporozoites. Further, from a mechanism of action viewpoint, the data may be adduced as evidence that militates against the mechanism of action model positing “activation” of artemisinins by heme or labile ferrous iron (Meunier and Robert, 2010), as heme metabolism in these latter stages is absent (Hanssen et al., 2012). The heme model invokes “toxic” C-radical intermediates that nevertheless are incapable of alkylating anything except perhaps an adjacent heme (spin-unpaired by virtue of the presence of its embedded redox-active iron), but that do react at a diffusion controlled rate with oxygen, itself a spin-unpaired triplet molecule (Haynes et al., 2013). On the other hand, we have noted that the optimum biological activities of amino-artemisinins as reported here may be accommodated within the model positing the oxidant mode of action for the artemisinins (Haynes et al., 2011, 2012) wherein the properties of the amino group at C-10 that contribute to oxidant capacity of the molecule are enhanced under physiological conditions (Haynes et al., 2016). Further, the marked synergism between artemisone and the redox active drug MB in the combination is indicative of disruption in redox homeostasis as a mechanism (Coertzen et al., 2018). In this respect, it is of particular relevance to note that primaquine is metabolized by CYP2D6 wherein a hydroxyl group is inserted in the 5-position, that is, in a para-relationship with the 8-amino group. The resulting amino-phenol undergoes facile oxidation to the quinone-imine by oxygen resulting in generation of ROS. In the presence of the flavoenzyme cytochrome P450 NADPH:oxidoreductase, the quinone-imine is reduced to the amino-phenol, and thus redox cycling ensues (Camarda et al., 2019). It is the ROS that exert cytotoxicity toward gametocytes; this redox cycling precisely mirrors the process as described above for methylene blue. Thus, it is likely that naphthoquinones and other redox-active reagents will behave in the same way, and should be active against gametocytes in a process that will be synergized by the amino-artemisinin. However, the exact mechanism by which gametocytes regulate their redox homeostasis in relation to that of asexual parasites remains to be established.

Our proposed new drug combinations of the amino-artemisinin with the redox active drug methylene blue, or a phenoxazine, naphthoquinone, metal-chelating agent or other as described above should offer a decisive advantage over the current ACTs that are ineffective in blocking transmission. Further, although MB is gametocytocidal (Adjalley et al., 2011), our newer amino-artemisinins are over 100-fold more active than MB toward late-stage gametocytes (Table 2). Because the amino-artemisinins are so potently gametocytocidal, the addition of other distinct gametocytocidal drugs, e.g., primaquine, in principle will not be required. In this respect, it is pertinent to note that primaquine is the critical transmission-blocking component used in mass drug administration (MDA) programmes being carried out purportedly to clear subclinical reservoirs of resistant parasites in humans in South-East Asia. These entail administration of a 3-day course of DHA-piperaquine plus single dose primaquine each month for 3 months to entire village populations including children over 6 months of age (Landier et al., 2018; von Seidlein et al., 2019). It should be of concern that the DHA-piperaquine appears to be the most fraught of all ACTs, given not just the problems with DHA alluded to above, but also because of resistance now emerging to the piperaquine component (Hamilton et al., 2019; WHO, 2019). Reduced efficacy of this ACT is also reflected in the need to apply protracted dose regimens to treat resistant parasites (Phyo and von Seidlein, 2017). In the face of these abnormally protracted treatment and MDA regimens, the earlier recommendation for increased pharmacovigilance, especially in a pediatric situation coupled with the issue of neurotoxicity with DHA (Ramos-Martín et al., 2014) would indeed appear to be appropriate.

Overall, given that activities of selected amino-artemisinins *in vitro* are not affected by current multidrug resistant strains (Tables 1, 2), and may be active against artemisinin-resistant *P. falciparum* phenotypes including those carrying the *Pf* KI3 C580Y mutation (Table 4), it is hoped that such activity will maintain *in vivo* thereby ensuring potent activity of the putative new ACTs against parasite strains of differing resistance profiles. Thus, to conclude, in addition to artemiside **7** and artemisone **8**, the best compounds for taking forward overall based on their efficacy, relative ease of preparation, and relative lack of toxicity are the aryl sulfonamide **16**, the aryl urea **23**, and the aryl amides **27** and **28**. Given especially their activities against late stage gametocytes, it is a matter of urgency to screen these compounds against in-mosquito stages of the malaria parasite (Vos et al., 2015).

## Data Availability Statement

All datasets generated for this study are included in the article/Supplementary Material.

## Ethics Statement

This study was carried out according the guidelines set out by the Faculty of Health Sciences ethical committee, ethical clearance no. EC-120821/077. The protocol was approved by the Faculty of Health sciences ethical committee at the University of Pretoria. All subjects gave written informed consent in accordance with the Declaration of Helsinki.

## Author Contributions

Inculcation of the concept and project overview was by RH. HW proposed additional structures and synthesized, characterized, and purified all the compounds. WL conducted the thermochemical studies and examined crystallinity of all compounds. DC, MW, JR, and L-MB conducted the efficacy assays (proliferative assay) with *Pf* NF54, K1, and W2 asexual parasites and NF54 gametocytes. LW and PS conducted efficacy assays (pLDH) with *Pf* NF54, Dd2 asexual parasites and toxicity assays. VP-I and DK conducted screens on the artemisinin-resistant strains. KE and EW conducted *Pb* liver stage and HepG2 cytotoxicity assays. RH and DC wrote the draft manuscript. All authors reviewed and provided input to generate the final version.

### Conflict of Interest

The authors declare that the research was conducted in the absence of any commercial or financial relationships that could be construed as a potential conflict of interest.

## Supplementary Material

Supplementary Table 1 *In vitro* activities of selected amino-artemisinins against liver stage *P. berghei*, dose response curves and cytotoxicities.

Supplementary Material comprises experimental details for synthesis and characterization data of the amino-artemisinins, and dose response curves for the *in vitro P. berghei* sporozoite stage efficacy assays recorded in Excel format in CDD Vault: UCSD CDD_Vault_Export_RESULTS_KDE_03-25-2019.
