# Supplementary material for: Optimal 10-Aminoartemisinins With Potent Transmission-Blocking Capabilities for New Artemisinin Combination Therapies–Activities Against Blood Stage P. falciparum Including PfKI3 C580Y Mutants and Liver Stage P. berghei Parasites
Source: Front Chem. 2020 Jan 10;7:901. doi: 10.3389/fchem.2019.00901 (PMC6967409; doi:10.3389/fchem.2019.00901)
Supplement: Supplementary file 1 [file Data_Sheet_1.docx]

Supplementary Material

# Synthetic Chemistry

**General**

Reagents were from Sigma–Aldrich, and used as supplied. Bulk solvents, magnesium sulfate and sodium hydrogen carbonate were from ACE Chemicals, Johannesburg, South Africa. Dichloromethane was distilled, dried over calcium carbonate and stored over 3Å molecular sieves. Diethyl ether and tetra­hydro­furan (THF) were dried over sodium, and distilled before use. Dihydroartemisinin (DHA) was pur­chased from Changzhou Kaixuan Chemical Co, Chunjiang, China and used as such. ^1^H and ^13^C NMR spectra were recorded on a Bruker Avance III spectrometer as solutions in CDCl_3_. Chemical shifts (δ) are reported in parts per million (ppm) and ^1^H chemical shifts are reported downfield of tetramethylsilane (TMS) with internal reference to the residual proton in CDCl_3_ (δ = 7.25 ppm). ^13^C chemical shifts were referenced to the CDCl_3_ resonances (δ = 77.00 ppm). The splitting patterns are abbreviated as follows: s (singlet), d (doublet), dd (doublet of doublets), t (triplet) and m (multiplet). The coupling constant *J* are reported in Hz. Spectra were analyzed with MestReNova Software, version 5.3.2–4936. High resolution mass spec­tro­­­­metry (HRMS) was recorded on a Bruker MicroTOF Q II mass spectrometer, equipped with an ESI source set at 180 °C using Bruker Compass DataAnalysis 4.0 software. Infrared (IR) spectra were recorded on a Bruker Alpha-P FTIR instrument. Melting points (mp) were determined on a Büchi melting point B-545 instrument, or via differential scanning calorimetry (DSC) using a DSC-60 Shimadzu instru­ment (Kyoto, Japan) to record the DSC thermograms. Samples weighing approximately 3-5 mg in aluminium crimp cells were heated to 250 °C with a heating rate of 10 °C/min, with a nitrogen gas flow of 35 mL/min; the DSC melting point was taken at the temperature corresponding to the peak of the exo­therm following onset of melting. Column chromato­­graphy was performed using high-purity-grade silica gel (pore size: 60 Å, 70–230 mesh, 63–200 µm) from Sigma–Aldrich and thin-layer chromatography (TLC) was performed with silica gel plates (60 F254) from Merck. For HPLC analyses, an Agilent 1100 series instrument equipped with a gradient pump, autosampler, Diode Array UV detector and OpenLab CDS Chemstation Rev.C.01.07 SR3 data acquisition and analysis software, and a Venusil XBP C18(2) column, 150 x 4.6 mm, 5 µm spherical particles, 100 Å pore size (Agela Technologies, Newark, DE, USA) were used. The mobile phase consisted of acetonitrile and 0.1% orthophosphoric acid in water with a linear gradient from 30% acetonitrile to 85% after five minutes and holding until 15 minutes before equilibrating with 30% acetonitrile to 20 minutes; for the derivatives, UV detection at UV at 210 nm was used. All compounds submitted for screening were demonstrated to be ≥95% pure, except where indicated.

**S.1.1 DHA-piperazine derivatives 13**-**28** (Scheme 1)

***DHA-Piperazine 9***

This has been prepared previously and fully characterized; it is a highly polar relatively less stable compound that is not readily purified (Coertzen, 2018; Wu, 2018). Thus, in order to expedite preparation of the derivatives here, the crude material isolated directly from the reaction mixture obtained from its preparation was used. Dimethyl sulfoxide (25.1 µL, 0.1 equiv.) was added into a stirred suspension of DHA (1.0 g, 3.517 mmol) in toluene (10 mL) at room temperature under nitrogen. Oxalyl chloride (0.35 mL, 1.15 equiv) was slowly added into the reaction mixture; during the addition the suspension became a clear amber-coloured solution. This was stirred for 1 hour, and then transferred via cannula into a stirred solution of piperazine (1.5 g, 5 equiv.) in dichloromethane (10 mL). The resulting mixture was stirred overnight, and then quenched with saturated aqueous NaHCO_3_ (20 mL). The mixture was extracted with ethyl acetate (4 x 30 mL), the extracts were combined and washed with brine (2 x 25 mL), and dried over MgSO_4_. The mixture was filtered, and the filtrate evaporated under reduced pressure to leave a semicrystalline residue, that was examined directly by ^1^H NMR spectroscopy. The product mixture consisted of DHA-piperazine **9** (H-12, s, δ = 5.27 ppm) admixed with the glycal by-product of this reaction (H-12, s, δ = 5.53 ppm) (Chan, 2018; Wu, 2018); the ratio was established by examining integrals of these signals as described in detail elsewhere for preparation of artemisone (Chan, 2018). The ratio of **9** to the glycal was 70:30, corresponding to the presence of 2.29 mmol of **9** in the residue; this ratio obtains for reactions run under identical conditions. As the glycal does not interfere, no further purification was carried out, and the mixture was used as such directly in the next step.

***General procedure for conversion of DHA-piperazine 9 into piperazine sulfonamides.***

A solution of the DHA-piperazine **9** (2.29 mmol) in THF (10 mL) under nitrogen was treated with triethyl­amine (1.2 equiv), and then the sulfonyl chloride (1.1 equiv). The reaction mixture was stirred for 20-24 hours at room temperature. It was quenched with saturated aqueous NH_4_Cl (20 mL). The mixture was extracted with ethyl acetate (3 x 20 mL), and the extracts were combined, washed with brine (20 mL) and dried over MgSO_4_. After filtration to remove the drying agent, the solvent was evaporated under reduced pressure to leave the residue, usually a microcrystalline solid or powder. The product was isolated by column chromatography on silica gel with ethyl acetate-hexane.

4'-*N*-Butanesulfonylpiperazine-DHA **13**: DHA-piperazine **9** (2.29 mmol) in THF (10 mL) under nitrogen was treated with triethyl­amine (0.38 mL, 1.2 equiv) and then with butanesulfonyl chloride 98% (0.33 mL, 1.1 equiv). The resulting mixture was stirred for 22 h at room temperature, and then processed as described above to leave the crude product as a semi-solid residue. The residues from two such reactions were combined and submitted to column chromatography with ethyl acetate-hexane 5:95 then 1:1 to give the product as a fine white powder (374 mg, 17%), DSC m.p. 136.1 °C. ^1^H NMR δ = 0.77 (3H, d, J = 7.2 Hz, 9-Me), 0.90-0.93 (6H, m, 6-Me and butyl-Me), 0.94-1.01 (1H, m), 1.20-1.32 (4H, m, 2 x butyl-CH_2_), 1.35 (3H, s, 3-Me), 1.39-1.45 (1H, m), 1.50-1.53 (1H, m), 1.66-1.70 (2H, m), 1.75-1.79 (2H, m), 1.81-1.85 (1H, m), 1.96-1.99 (1H, m), 2.29-2.34 (1H, m), 2.52-2.57 (1H, m), 2.71-2.75 (2H, m, piperazine), 2.86-2.88 (2H, m, butyl-CH_2_), 3.00-3.04 (2H, m, piperazine), 3.22-3.27 (4H, m, piperazine ), 4.01 (1H, d, J = 10.2 Hz, H-10), 5.24 (1H, s, H-12); ^13^C NMR δ 13.42, 13.55, 20.23, 21.57, 21,72, 24.70, 25.00, 25.92, 28.41, 34.20, 36.22, 37.34, 45.71, 45.95, 48.99, 51.62, 80.20, 90.62, 91.51, 103.93. IR (film): ν_max_ = 510, 537, 550, 576, 605, 716, 738, 777, 792, 826, 851, 879, 924, 952, 979, 1020, 1041, 1061, 1085, 1100, 1115, 1130, 1146, 1180, 1204, 1262, 1278, 1323, 1338, 1378, 1454, 2858, 2872, 2927 cm^-1^. MS *m/z* calcd for C_23_H_41_N_2_O_6_S^+^ 473.2685 [M+H]^+^; found 473.2671.

4'-*N*-Hexadecanesulfonylpiperazine-DHA **14**: DHA-piperazine **9** (2.29 mmol) in THF (10 mL) containing triethylamine (0.38 ml, 1.2 equiv) was treated with a solution of hexadecanesulfonyl chloride (98%, 835 mg, 1.1 equiv) in THF (5mL), and the resulting mixture was stirred for 22 h at room tem­perature. The reaction mixture was quenched and worked up as described above to leave a solid residue that was submitted to chromatography with ethyl acetate-hexane 5:95 then 1:1 to give the product (852 mg, 58%) as a powder, DSC m.p. 132.7 ^o^C. ^1^H NMR δ = 0.76 (3H, d, J = 7.2 Hz, 9-Me), 0.84 (3H, t, J = 7.2 Hz, hexadecyl-Me), 0.91 (3H, d, J = 6.0 Hz, 6-Me), 0.94-1.01 (1H, m), 1.22-1.26 (28H, m, 14 x hexadecyl-CH_2_), 1.35 (3H, s, 3-Me), 1.42-1.45 (1H, m), 1.50-1.53 (1H, m), 1.65-1.69 (2H, m), 1.75-1.79 (2H, m), 1.81-1.86 (1H, m), 1.95-1.99 (1H, m), 2.28-2.34 (1H, m), 2.52-2.57 (1H, m), 2.71-2.74 (2H, m, piperazine), 2.84-2.87 (2H, m, hexadecyl-CH_2_), 3.00-3.04 (2H, m, piperazine), 3.21-3.26 (4H, m, piperazine), 4.00-4.02 (1H, d, J = 10.2 Hz, H-10), 5.24 (1H, s, H-12); ^13^C NMR δ = 13.41, 14.08, 20.22, 21.55, 22.64, 23.00, 24.69, 28.40, 28.49, 29.06, 29.27, 29.31, 29.47, 29.54, 29.60, 29.64, 31.87, 34.19, 36.21, 37.33, 45.69, 45.94, 49.19, 51.60, 80.18, 90.59, 91.49, 103.91. IR (film) ν_max_ = 540, 721, 786, 796, 827, 851, 878, 925, 953, 963, 979, 1024, 1044, 1065, 1100, 1114, 1130, 1147, 1161, 1205, 1310, 1325, 1339, 1468, 2848, 2919 cm^-1^. MS *m/z* calcd for C_35_H_65_N_2_O_6_S^+^ 641.4563 [M+H]^+^; found 641.4512.

4'-*N*-(*p*-Acetamidobenzenesulfonyl)piperazine-DHA **15**: DHA-piperazine **9** (2.29 mmol) in THF (10 mL) under nitrogen containing triethylamine (0.38 mL, 1.2 equiv) was treated with a solution of 4-acetamidobenzenesulfonyl chloride (98%, 601 mg, 1.1 equiv) in THF (5 mL), and the resulting mixture was stirred for 22 h at room temperature. The reaction mixture was quenched and worked up as described above to leave a solid residue that after chromatography with ethyl acetate-hexane 1:1 gave the product (690 mg, 53%) as a powder, DSC m.p. 145.9 ^o^C. ^1^H NMR δ = 0.66 (3H, d, J = 6.8 Hz, 9-Me), 0.89 (3H, d, J = 6.5 Hz, 6-Me), 0.94-1.01 (1H, m), 1.32 (3H, s, 3-Me), 1.38-1.43 (1H, m), 1.45-1.48 (1H, m), 1.61-1.65 (2H, m), 1.80-1.84 (1H, m), 1.94-1.97 (1H, m), 2.17 (3H, s, Me, 4-acetamide), 2.26-2.32 (1H, m), 2.45-2.48 (1H, m), 2.70-2.72 (2H, m, piperazine), 2.93-3.01 (6H, m, piperazine), 3.93 (1H, d, J = 10.2 Hz, H-10), 5.20 (1H, s, H-12), 7.64 (2H, d, J = 9.1 Hz, ArH), 7.68 (2H, d, J = 8.8 Hz, ArH), 8.15 (1H, s, N-H); ^13^C NMR δ = 13.32, 14.12, 20.19, 21.01, 21.50, 24.58, 24.66, 25.68, 28.36, 34.13, 36.18, 37.29, 45.65, 46.31, 51.57, 60.38, 80.13, 90.24, 91.39, 103.81, 119.27, 128.79, 142.51, 169.29. IR (film) ν_max_ = 404, 444, 472, 485, 510, 525, 549, 573, 611, 634, 700, 742, 828, 850, 881, 894, 925, 942, 982, 1025, 1041, 1061, 1101, 1116, 1129, 1162, 1181, 1205, 1230, 1262, 1271, 1324, 1345, 1375, 1400, 1452, 1496, 1532, 1593, 1674, 1993, 2178, 2865, 2922, 3051, 3108, 3309 cm^-1^. MS *m/z* calcd for C_27_H_41_N_4_O_7_S^+^ 550.2587 [M+H]^+^; found 550.2587.

4'-*N*-(*m*-Trifluoromethylbenzenesulfonyl)piperazine-DHA **16**: DHA-piperazine **9** (2.29 mmol) in THF (10 mL) under nitrogen containing triethylamine (0.38 mL, 1.2 equiv) was treated with 3-(trifluoro­methyl)­­benzene­sulfonyl chloride (95%, 0.43 mL, 1.1 equiv). and the resulting mixture was stirred for 22 h at room temperature. The reaction mixture was quenched and worked up as described above to leave a solid residue that after chromatography with ethyl acetate-hexane 1:3 gave the product (739 mg, 58%) as a powder, DSC m.p. 140.2 ^o^C. ^1^H NMR δ = 0.67-0.68 (3H, d, J = 7.1 Hz, 9-Me), 0.90-0.91 (3H, d, J = 6.5 Hz, 6-Me), 0.94-0.99 (1H, m), 1.31 (3H, s, 3-Me), 1.41-1.43 (1H, m), 1.46-1.50 (1H, m), 1.62-1.65 (2H, m), 1.80-1.84 (1H, m), 1.94-1.98 (1H, m), 2.28-2.32 (1H, m), 2.46-2.50 (1H, m), 2.73-2.76 (2H, m, piperazine), 3.01-3.05 (6H, m, piperazine ), 3.95 (1H, d, J = 10.1 Hz, H-10), 5.21 (1H, s, H-12), 7.67 (1H, t, J = 7.9 Hz, ArH), 7.83 (1H, d, J = 8 Hz, ArH), 7.93 (1H, d, J = 7.9 Hz, ArH), 7.99 (1H, s, ArH); ^13^C NMR δ = 13.30, 20.18, 21.49, 24.66, 25.83, 28.35, 34.13, 36.16, 37.29, 45.63, 46.20, 51.55, 80.12, 90.35, 91.42, 103.88, 129.79, 130.89, 137.44. IR (film) ν_max_ = 474, 485, 512, 550, 564, 577, 651, 697, 736, 808, 825, 851, 879, 895, 926, 953, 983, 1025, 1041, 1087, 1102, 1127, 1168, 1203, 1263, 1280, 1307, 1326, 1357, 1376, 1453, 2871, 2926 cm^-1^. MS *m/z* calcd for C_26_H_36_F_3_N_2_O_6_S^+^ 561.2246 [M+H]^+^; found 561.2260.

4'-*N*-(*m*-Fluorobenzenesulfonyl)piperazine-DHA **17**: DHA-piperazine **9** (2.29 mmol) in THF (10 mL) under nitrogen containing triethylamine (0.38 mL, 1.2 equiv) was treated with 3-fluorobenzenesulfonyl chloride 97% (0.35 mL, 1.1 equiv) and the resulting mixture was stirred for 23 h at room temperature. The reaction mixture was quenched and worked up as described above to leave a solid residue that after chromatography with ethyl acetate-hexane 1:3 gave the product (609 mg, 52%) as a fine powder, DSC m.p. 135.7 °C. ^1^H NMR δ = 0.69 (3H, d, J = 7.2 Hz, 9-Me), 0.92 (3H, d, J = 6.1 Hz, 6-Me), 0.94-0.99 (1H, m), 1.33 (3H, s, 3-Me), 1.38-1.44 (1H, m), 1.47-1.50 (1H, m), 1.64-1.65 (2H, m), 1.81-1.84 (1H, m), 1.96-1.98 (1H, m), 2.28-2.33 (1H, m), 2.47-2.51 (1H, m), 2.73-2.74 (2H, m, piperazine), 3.00-3.04 (6H, m, piperazine), 3.95 (1H, d, J = 10.2 Hz, H-10), 5.21 (1H, s, H-12), 7.26-7.28 (1H, m, ArH), 7.44-7.54 (3H, m, ArH); ^13^C NMR δ = 13.34, 20.22, 21.54, 24.71, 25.88, 28.39, 34.19, 36.22, 37.34, 45.69, 46.29, 51.61, 80.16, 90.43, 91.46, 103.93, 114.95, 115.11, 119.78, 119.92, 123.49, 123.51, 130.72, 130.77, 138.22, 138.27, 161.58, 163.25. IR (film) ν_max_ = 474, 485, 517, 533, 552, 578, 611, 682, 729, 793, 827, 850, 879, 925, 943, 982, 1025, 1040, 1085, 1099, 1128, 1167, 1205, 1223, 1271, 1303, 1327, 1351, 1376, ,1434, 1453, 1474, 2590, 2869, 2925 cm^-1^. MS *m/z* calcd for C_25_H_36_FN_2_O_6_S^+^ 511.2278 [M+H]^+^; found 511.2272.

4'-*N*-(*p*-Trifluoromethylbenzenesulfonyl)piperazine-DHA **18**: DHA-piperazine **9** (2.29 mmol) in THF (10 mL) under nitrogen containing triethylamine (0.38 mL, 1.2 equiv) was treated with a solution of 4-(trifluoro­methyl)benzenesulfonyl chloride (97%, 635 mg, 1.1 equiv) in THF (5 mL) and the resulting mixture was stirred for 22 h at room tem­perature. The reaction mixture was quenched and worked up as described above to leave a solid micro­crystalline residue, attempted chromatography of which resulted in considerable loss of material. Therefore, the residue was dissolved in ethyl acetate (20 mL), concentrative evaporation of which induced precipitation of the product as a powder. This was collected by filtration and was washed successively with ethyl acetate-hexane (1:9, 50 mL) and ethyl acetate-hexane (3:7, 25 mL), and then dried under vacuum to leave the product as a fine powder (337 mg, 26%), DSC m.p. 146.65 °C. ^1^H NMR δ = 0.68 (3H, d, J = 7.2 Hz, 9-Me), 0.91 (3H, d, J = 6.1 Hz, 6-Me), 0.94-0.99 (1H, m), 1.30 (3H, s, 3-Me), 1.38-1.44 (1H, m), 1.47-1.50 (1H, m), 1.64-1.65 (2H, m), 1.81-1.85 (1H, m), 1.95-1.99 (1H, m), 2.27-2.33 (1H, m), 2.47-2.50 (1H, m), 2.75-2.76 (2H, m, piper­azine), 3.03-3.05 (6H, m, piperazine), 3.96 (1H, d, J = 10.1 Hz, H-10), 5.21 (1H, s, H-12), 7.78 (2H, d, J = 8.3 Hz, ArH), 7.87 (2H, d, J = 8.2 Hz, ArH); ^13^C NMR δ = 13.36, 20.23, 21.55, 24.70, 25.86, 28.41, 34.17, 36.19, 37.35, 45.67, 46.23, 51.59, 80.17, 90.47, 91.45, 103.95, 126.15, 128.21. IR (film) ν_max_ = 429, 472, 486, 550, 566, 595, 611, 699, 727, 742, 787, ,911, 827, 843, 862, 880, 895, 926, 944, 984, 1014, 1025, 1060, 1104, 1124, 1172, 1250, 1261, 1271, 1301, 1322, 1348, 1356, 1378, 1402, 1455, 2863, 2926, 2972 cm^-1^. MS *m/z* calcd for C_26_H_36_F_3_N_2_O_6_S^+^ 561.2246 [M+H]^+^; found 561.2223.

4'-*N*-(*p*-Fluorobenzenesulfonyl)piperazine-DHA **19**: DHA-piperazine **9** (2.29 mmol) in THF (10 mL) under nitrogen containing triethylamine (0.38 mL, 1.2 equiv) was treated with a solution of 4-fluorobenzenesulfonyl chloride (98%, 500 mg, 1.1 equiv) in THF (5 mL), and the resulting mixture was stirred for 22 h at room tem­perature. The reaction mixture was worked up as described above to leave the residue that was dissolved in ethyl acetate (30 mL). As attempts to chromatograph the material resulted in considerable loss of material, concentrative evaporation of the ethyl acetate solution was used to induce precipitation of the product as a powder. This was collected by filtration and was washed success­ively with ethyl acetate-hexane (1:9, 50 mL) and ethyl acetate-hexane (3:7, 25 mL), and then dried under vacuum. The product (318 mg, 27%) was obtained as a fine powder, DSC m.p. 127.65 °C. ^1^H NMR δ = 0.68 (3H, d, J = 7.3 Hz, 9-Me), 0.91 (3H, d, J = 5.9 Hz, 6-Me), 0.94-0.99 (1H, m), 1.32 (3H, s, 3-Me), 1.39-1.44 (1H, m), 1.46-1.49 (1H, m), 1.63-1.65 (2H, m), 1.81-1.84 (1H, m), 1.95-1.99 (1H, m), 2.27-2.33 (1H, m), 2.47-2.50 (1H, m), 2.73-2.74 (2H, m, piperazine), 2.97-3.03 (6H, m, piperazine), 3.95 (1H, d, J = 10.2 Hz, H-10), 5.21 (1H, s, H-12), 7.17-7.20 (2H, m, ArH), 7.74-7.77 (2H, m, ArH); ^13^C NMR δ = 13.34, 20.22, 21.53, 24.69, 25.86, 28.38, 34.16, 36.19, 37.33, 45.66, 46.25, 51.58, 80.16, 90.39, 91.45, 103.92, 116.14, 116.29, 130.38, 130.44. IR (film) ν_max_ = 474, 487, 510, 528, 545, 575, 605, 655, 708, 730, 748, 817, 837, 862, 880, 924, 950, 984, 1024, 1045, 1064, 1097, 1127, 1157, 1174, 1202, 1225, 1262, 1271, 1297, 1326, 1344, 1354, 1380, 1454, 1492, 1590, 2854, 2923 cm^-1^. MS *m/z* calcd for C_25_H_36_FN_2_O_6_S^+^ 511.2278 [M+H]^+^; found 511.2260.

***General procedure for conversion of DHA-piperazine 9 into piperazine ureas.***

A solution of DHA-piperazine **9** (2.29 mmol) in dry dichloromethane (12 mL) under nitrogen was treated with isocyanate (1 equiv) at room temperature, and the resulting solution was stirred for 24 hours. The reaction mixture was concentrated directly by evaporation under reduced pressure to leave the residue that was submitted to column chromatography on silica gel with ethyl acetate-hexane to isolate the product.

4'-*N*-(Hexylaminocarbonyl)piperazine-DHA **20**: According to the foregoing procedure, DHA-piperazine **9** (2.29 mmol) in dichloromethane (12 mL) under nitrogen was treated with hexyl isocyanate (97%, 0.35 mL, 1 equiv) and the resulting solution was stirred for 24 h at room temperature. The reaction mixture was then concentrated by direct evaporation under reduced pressure to leave a solid residue that after chromatography with ethyl acetate-hexane 3:7 then 1:1 gave the product (765 mg, 70%) as a powder, DSC m.p. 118.6 °C. ^1^H NMR δ = 0.79 (3H, d, J = 7.1 Hz, 9-Me), 0.86 (3H, t, J = 7.1 Hz, hexyl-Me), 0.93 (3H, d, J = 6.3 Hz, 6-Me), 0.94-1.01 (1H, m), 1.25-1.31 (8H, m, 4 x hexyl-CH_2_), 1.36 (3H, s, 3-Me), 1.43-1.47 (1H, m), 1.51-1.53 (1H, m), 1.66-1.70 (2H, m), 1.82-1.85 (1H, m), 1.97-1.99 (1H, m), 2.29-2.34 (1H, m), 2.54-2.57 (1H, m), 2.61-2.64 (2H, m, piperazine), 2.94-2.96 (2H, m, piperazine), 3.17-3.20 (2H, m, hexyl-CH_2_), 3.31 (4H, m, piperazine), 4.01 (1H, d, J = 10.2 Hz, H-10), 4.36 (1H, s, N-H), 5.25 (1H, s, H-12); ^13^C NMR δ = 13.46, 14.03, 20.27, 21.60, 22.57, 24.72, 25.96, 26.62, 28.44, 30.23, 31.55, 34.24, 36.27, 37.37, 40.93, 44.09, 45.78, 51.67, 80.28, 90.03, 91.59, 103.91. IR (film) ν_max_ = 422, 442, 486, 510, 552, 564, 600, 616, 694, 727, 744, 769, 824, 834, 853, 881, 895, 922, 943, 958, 983, 1009, 1026, 1044, 1060, 1086, 1105, 1115, 1136, 1148, 1157, 1187, 1197, 1213, 1231, 1260, 1287, 1312, 1338, 1380, 1422, 1456, 1520, 1637, 1727, 2183, 2853, 2924, 2952, 2978, 3420 cm^-1^. MS *m/z* calcd for C_26_H_46_N_3_O_5_^+^ 480.3437 [M+H]^+^; found 480.3418.

4'-*N*-(Hexadecylaminocarbonyl)piperazine-DHA **21**: According to the foregoing procedure, DHA-piper­azine **9** (2.29 mmol) in dichloromethane (12 mL) under nitrogen was treated with hexadecyl isocyanate (97%, 0.73 mL, 1 equiv) and the resulting solution stirred for 24 h at room temperature. The reaction mixture was then concentrated by direct evaporation under reduced pressure to leave a solid residue that after chromatography with ethyl acetate-hexane 3:7 then 1:1 gave the product (978 mg, 69%) as a powder, DSC m.p. 129.3 °C. ^1^H NMR δ = 0.79 (3H, d, J = 6.3 Hz, 9-Me), 0.85 (3H, t, J = 7.1 Hz, hexadecyl Me), 0.93 (3H, d, J = 6.0 Hz, 6-Me), 0.94-1.01 (1H, m), 1.22-1.26 (28H, m, 14 x hexadecyl-CH_2_), 1.35 (3H, s, 3-Me), 1.43-1.47 (1H, m), 1.50-1.53 (1H, m), 1.64-1.70 (2H, m), 1.82-1.85 (1H, m), 1.96-1.99 (1H, m), 2.29-2.34 (1H, m), 2.54-2.57 (1H, m), 2.61-2.64 (2H, m, piperazine), 2.94-2.96 (2H, m, piperazine), 3.17-3.20 (2H, m, hexadecyl-CH_2_), 3.31 (4H, m, piperazine), 4.01 (1H, d, J = 9.5 Hz, H-10), 4.36 (1H, s, N-H), 5.24 (1H, s, H-12); ^13^C NMR δ = 13.46, 14.11, 20.27, 21.60, 22.67, 24.72, 25.96, 26.97, 28.44, 29.35, 29.38, 29.57, 29.60, 29.64, 29.68, 31.91, 34.24, 36.26, 37.37, 40.94, 44.09, 45.78, 51.67, 80.27, 90.63, 91.58, 103.91. IR (film) ν_max_ = 425, 437, 465, 488, 510, 529, 544, 553, 698, 718, 754, 808, 828, 849, 860, 878, 894, 911, 926, 944, 958, 980, 1022, 1041, 1055, 1084, 1116, 1134, 1145, 1157, 1187, 1197, 1210, 1257, 1279, 1312, 1335, 1355, 1373, 1406, 1452, 1470, 1522, 1633, 1724, 2850, 2918, 3453 cm^-1^. MS *m/z* calcd for C_36_H_66_N_3_O_5_^+^ 620.5002 [M+H]^+^; found 620.4955.

4'-*N*-(p-*tert*-Butylphenylaminocarbonyl)piperazine-DHA **22**: DHA-piperazine **9** (2.29 mmol) in dichloro­methane (12 mL) under nitrogen was treated with 4-*tert*-butylphenyl isocyanate (97%, 0.42 mL, 1 equiv) and the resulting solution stirred for 24 h at room temperature. The reaction mixture was then concentrated by direct evaporation under reduced pressure to leave a solid residue that after chromatography with ethyl acetate-hexane 3:7 then 1:1 gave the product (814 mg, 67%) as a powder, DSC m.p. 160.0 °C. ^1^H NMR δ = 0.81 (3H, d, J = 7.3 Hz, 9-Me), 0.93 (3H, d, J = 6.5 Hz, 6-Me), 0.95-1.02 (1H, m), 1.26, (9H, s, *tert*-butyl), 1.36 (3H, s, 3-Me), 1.43-1.46 (1H, m), 1.52-1.54 (1H, m), 1.66-1.71 (2H, m), 1.82-1.86 (1H, m), 1.97-1.99 (1H, m), 2.29-2.34 (1H, m), 2.54-2.59 (1H, m), 2.68-2.70 (2H, m, piperazine), 2.99-3.01 (2H, m, piperazine), 3.44 (4H, m, piperazine), 4.03 (1H, d, J = 10.3 Hz, H-10), 5.25 (1H, s, H-12), 6.30 (1H, s, N-H), 7.22-7.27 (4H, m, ArH); ^13^C NMR δ = 13.46, 20.25, 21.59, 24.70, 25.96, 28.43, 31.37, 34.18, 34.21, 36.25, 37.35, 44.40, 45.75, 51.64, 80.26, 90.60, 91.57, 103.93, 119.75, 125.63, 136.34, 145.80, 155.13. IR (film) ν_max_ = 427, 439, 458, 469, 487, 517, 552, 625, 696, 717, 740, 829, 846, 879, 893, 924, 942, 959, 996, 1029, 1045, 1059, 1085, 1107, 1118, 1132, 1158, 1197, 1208, 1239, 1269, 1284, 1315, 1351, 1379, 1417, 1448, 1509, 1592, 1656, 2844, 2922, 2944, 3420 cm^-1^. MS *m/z* calcd for C_30_H_46_N_3_O_5_^+^ 528.3437 [M+H]^+^; found 528.3444.

4'-*N*-(*p*-Trifluoromethylphenylaminocarbonyl)piperazine-DHA **23**: DHA-piperazine **9** (2.29 mmol) in dichloro­­methane (12 mL) under nitrogen was treated with 4-(trifluoro­methyl)phenyl iso­cyanate (99%, 0.33 mL, 1 equiv) and the resulting solution stirred for 24 h at room temperature. The reaction mixture was then concentrated by direct evaporation under reduced pressure to leave the solid residue that after chromatography with ethyl acetate-hexane 3:7 then 1:1 gave the product (770 mg, 62%) as small needles, DSC m.p. 158.55 °C. ^1^H NMR δ = 0.81 (3H, d, J = 6.5 Hz, 9-Me), 0.94(3H, d, J = 6.0 Hz, 6-Me), 0.96-1.03 (1H, m), 1.34 (1H, s, 3-Me), 1.41-1.48 (1H, m), 1.52-1.55 (1H, m), 1.67-1.71 (2H, m), 1.83-1.86 (1H, m), 1.97-2.00 (1H, m), 2.30-2.35 (1H, m), 2.57-2.59 (1H, m), 2.69-2.71 (2H, m, piperazine), 3.00-3.02 (2H, m, piperazine), 3.48 (4H, m, piperazine), 4.04 (1H, d, J = 9.87 Hz, H-10), 5.26 (1H, s, H-12), 6.52 (1H, s, N-H), 7.45-7.46 (2H, m, ArH), 7.49-7.51 (2H, m, ArH); ^13^C NMR δ = 13.36, 20.25, 21.59, 24.70, 25.97, 28.44, 34.21, 36.25, 37.37, 44.47, 45.74, 51.65, 80.28, 90.61, 91.60, 104.00, 118.95, 126.08, 142.30, 154.20. IR (film) ν_max_ = 441, 486, 504, 529, 592, 619, 637, 663, 694, 720, 740, 832, 847, 863, 883, 895, 926, 941, 959, 988, 1027, 1059, 1084, 1121, 1156, 1186, 1210, 1242, 1290, 1314, 1330, 1353, 1382, 1399, 1424, 1438, 1454, 1510, 1527, 1598, 1616, 1685, 2847, 2873, 2917, 2958, 3409 cm^-1^. MS *m/z* calcd for C_27_H_37_F_3_N_3_O_5_^+^ 540.2685 [M+H]^+^; found 540.2693. Analysis by HPLC indicates a purity of 99%.

4'-*N*-(*p*-Fluorophenylaminocarbonyl)piperazine-DHA **24**: DHA-piperazine **9** (2.29 mmol) in dichloro­methane (12 mL) under nitrogen was treated with 4-fluoro­phenyl isocyanate (98%, 0.27 mL, 1 equiv) and the resulting solution stirred for 24 h at room temperature. The reaction mixture was then concentrated by direct evaporation under reduced pressure to leave a solid residues that after chromatography with ethyl acetate-hexane 3:7 then 1:1 gave the product (967 mg, 86%) as a powder, DSC m.p. 139.6 °C. ^1^H NMR δ = 0.81 (3H, d, J = 6.8 Hz, 9-Me), 0.93 (3H, d, J = 6.5 Hz, 6-Me), 0.95-1.03 (1H, m), 1.34 (3H, s, 3-Me), 1.43-1.46 (1H, m), 1.52-1.54 (1H, m), 1.64-1.71 (2H, m), 1.83-1.86 (1H, m), 1.97-1.99 (1H, m), 2.29-2.35 (1H, m), 2.57-2.59 (1H, m), 2.69-2.70 (2H, m, piperazine), 3.00-3.01 (2H, m, piperazine), 3.44 (4H, m, piperazine), 4.04 (1H, d, J = 10.1 Hz, H-10), 5.26 (1H, s, H-12), 6.33 (1H, s, N-H), 6.93-6.96 (2H, m, ArH), 7.26-7.28 (2H, m, ArH); ^13^C NMR δ = 13.46, 20.26, 21.60, 24.70, 25.97, 28.44, 34.21, 36.25, 37.36, 44.38, 45.74, 51.65, 80.28, 90.61, 91.60, 103.96, 115.30, 115.44, 121.90, 155.02. IR (film) ν_max_ = 457, 467, 494, 510, 520, 553, 632, 698, 732, 746, 778, 825, 849, 860, 879, 926, 943, 956, 979, 1000, 1023, 1041, 1053, 1085, 1100, 1116, 1131, 1156, 1209, 1255, 1284, 1300, 1331, 1352, 1376, 1400, 1430, 1470, 1509, 1528, 1610, 1638, 1740, 2868, 2923, 2960, 3263 cm^-1^. MS *m/z* calcd for C_26_H_37_FN_3_O_5_^+^ 490.2717 [M+H]^+^; found 490.2760. Analysis by HPLC indicates a purity of 99%.

***General procedure for conversion of DHA-piperazine 9 into piperazine amides.***

A solution of the DHA-piperazine **9** (2.29 mmol) in THF (10-12 mL) under nitrogen was treated with triethyl­amine (1.2 equiv) and then the sulfonyl chloride (1.1 equiv). The reaction mixture was stirred for 20-24 hours at room temperature. It was quenched with saturated aqueous NH_4_Cl (20 mL). The mixture was extracted with ethyl acetate (3 x 20 mL), and the extracts were combined and washed with brine (20 mL) and dried over MgSO_4_. After filtration to remove the drying agent, the filtrate was evaporated under reduced pressure to leave the residue, usually a microcrystalline solid or powder. The product was isolated by column chromatography on silica gel with ethyl acetate-hexane.

4'-*N*-Hexanoylpiperazine-DHA **25**: DHA-piperazine **9** (2.29 mmol) in THF (12 mL) under nitrogen containing triethylamine (0.38 mL, 1.2 equiv) was treated with hexanoyl chloride (99%, 0.36 mL, 1.1 equiv), and the resulting mixture was stirred for 23 h at room temperature. The reaction mixture was quenched and worked up as described above to leave a solid residue that after chromatography with ethyl acetate-hexane 3:7 then 1:1 gave the product (695 mg, 67%) as a powder, DSC m.p. 128.5 °C. ^1^H NMR δ = 0.80 (3H, d, J = 6.8 Hz, 9-Me), 0.87 (3H, t, J = 7.3 Hz, hexanoyl-Me), 0.93 (3H, d, J = 6.5 Hz, 6-Me), 0.95-1.02 (1H, m), 1.18-1.23 (1H, m), 1.29-1.33 (6H, m, 3 x hexanoyl-CH_2_), 1.36 (3H, s, 3-Me), 1.41-1.48 (1H, m), 1.51-1.53 (1H, m), 1.57-1.61 (3H, m), 1.67-1.70 (2H, m), 1.83-1.85 (1H, m), 1.97-1.99 (1H, m), 2.27-2.34 (1H, m, 2H, m, hexanoyl-CH_2_), 2.56-2.62 (2H, m, piperazine), 2.93-3.00 (2H, m, piperazine), 3.38-3.44 (2H, m, piperazine), 3.57 (2H, m, piperazine), 4.01 (1H, d, J = 10.2 Hz, H-10), 5.25 (1H, s, H-12); ^13^C NMR δ = 13.45, 13.96, 20.26, 21.60, 22.47, 24.71, 25.09, 25.96, 28.42, 31.68, 33.32, 34.23, 36.26, 37.36, 41.81, 45.77, 51.68, 80.25, 90.62, 91.61, 103.95, 171.60. IR (film) ν_max_ = 487, 509, 536, 553, 620, 698, 716, 744, 764, 807, 828, 851, 880, 894, 925, 943, 979, 1020, 1038, 1085, 1102, 1115, 1132, 1157, 1187, 1197, 1209, 1227, 1237, 1252, 1278, 1310, 1330, 1351, 1376, 1434, 1448, 1631, 2860, 2921, 2953 cm^-1^. MS *m/z* calcd for C_25_H_43_N_2_O_5_^+^ 451.3172 [M+H]^+^; found 451.3164.

4'-*N*-Hexadecanoylpiperazine-DHA **26**: DHA-piperazine **9** (2.29 mmol) in THF (12 mL) under nitrogen containing triethylamine (0.38 mL, 1.2 equiv) was treated with hexadecanoyl chloride (98%, 0.78 mL, 1.1 equiv), and the resulting mixture was stirred for 23 h at room temperature. The reaction mixture was quenched and worked up as described above to leave a solid residue that after chromato­­graphy with ethyl acetate-hexane 3:7 gave the product (676.5 mg, 50%) as a powder, DSC m.p. 146.3 °C. ^1^H NMR δ = 0.80 (3H, d, J = 7.3 Hz, 9-Me), 0.86 (3H, t, J = 7.2 Hz, hexadecanoyl-Me), 0.93 (3H, d, J = 6.5 Hz, 6-Me), 0.95-1.01 (1H, m), 1.18-1.31 (26H, m, 13 x hexadecanoyl-CH_2_), 1.36 (3H, s, 3-Me), 1.41-1.48 (1H, m), 1.51-1.53 (1H, m), 1.57-1.60 (3H, m), 1.66-1.70 (2H, m), 1.82-1.86 (1H, m), 1.97-1.99 (1H, m), 2.26-2.34 (1H, m, 2H, m, hexadecanoyl-CH_2_), 2.55-2.62 (2H, m, piperazine), 2.91-2.95 (2H, m, piperazine), 3.38-3.44 (2H, m, piperazine) 3.57 (2H, m, piperazine), 4.01 (1H, d, J = 10.2 Hz, H-10), 5.25 (1H, s, H-12); ^13^C NMR δ = 13.46, 14.12, 20.26, 21.60, 22.68, 24.72, 25.41, 25.97, 28.42, 29.35, 29.44, 29.52, 29.64, 29.67, 31.91, 33.40, 34.24, 36.27, 37.37, 41.80, 45.77, 45.98, 51.68, 80.26, 90.63, 91.60, 103.95, 171.61. IR (film) ν_max_ = 488, 510, 538, 554, 624, 698, 721, 746, 782, 809, 826, 852, 881, 895, 926, 943, 958, 981, 1020, 1040, 1055, 1086, 1106, 1133, 1158, 1188, 1198, 1210, 1233, 1252, 1278, 1312, 1329, 1376, 1435, 1447, 1466, 1632, 2850, 2919 cm^-1^. MS *m/z* calcd for C_35_H_63_N_2_O_5_^+^ 591.4737 [M+H]^+^; found 591.4730

4'-*N*-(*p*-Trifluoromethylbenzoyl)piperazine-DHA **27**: DHA-piperazine **9** (2.29 mmol) in THF (12 mL) under nitrogen containing triethylamine (0.38 mL, 1.2 equiv) was treated with neat 4-trifluoromethylbenzoyl chloride (97%, 0.38 mL, 1.1 equiv), and the resulting mixture was stirred for 23 h at room temperature. The reaction mixture was quenched and worked up as described above to leave a solid residue that after chromatography with ethyl acetate-hexane 3:7 then 1:1 gave the product (917 mg, 76%) as micro­crystalline plates, DSC m.p. 153.1 °C. ^1^H NMR: δ = 0.79 (3H, d, J = 7.2 Hz, 9-Me), 0.92 (3H, d, J = 6.5 Hz, 6-Me), 0.94-1.01 (1H, m), 1.18-1.24 (2H, m), 1.28-1.34 (2H, m), 1.38 (3H, s, 3-Me), 1.40-1.47 (1H, m), 1.50-1.53 (1H, m), 1.65-1.70 (2H, m), 1.82-1.85 (1H, m), 1.97-1.99 (1H, m), 2.29-2.34 (1H, m), 2.52-2.60 (2H, m, piperazine), 2.92-3.06 (2H, m, piperazine) 3.31-3.33 (2H, m, piperazine), 3.68-3.81, (2H, m, piperazine), 4.01 (1H, d, J = 10.2 Hz, H-10), 5.25 (1H, s, H-12), 7.49 (2H, d, J = 7.9 Hz, ArH), 7.64 (2H, d, J = 8.0 Hz, ArH); ^13^C NMR δ = 13.41, 20.21, 21.56, 24.66, 25.96, 28.38, 34.17, 36.22, 37.32, 45.70, 51.64, 80.20, 90.63, 91.63, 103.98, 125.46, 125.49, 127.41, 168.68. IR (film) ν_max_ = 442, 613, 767, 827, 850, 879, 898, 926, 943, 981, 1013, 1041, 1063, 1124, 1161, 1188, 1207, 1262, 1280, 1323, 1376, 1406, 1435, 1637, 2871, 2925 cm^-1^. MS *m/z* calcd for C_27_H_36_F_3_N_2_O_5_^+^ 525.2576 [M+H]^+^; found 525.2591. Analysis by HPLC indicates a purity of 99%.

4'*-N-*(*p*-Fluorobenzoyl)piperazine-DHA **28**: DHA-piperazine **9** (2.29 mmol) in THF (12 mL) under nitrogen containing triethylamine (0.38 mL, 1.2 equiv) was treated with neat 4-fluorobenzoyl chloride (98%, 0.3 mL, 1.1 equiv), and the resulting mixture was stirred for 23 h at room temperature. The reaction mixture was quenched and worked up as described above to leave a solid residue that after chromato­graphy with ethyl acetate-hexane 3:7 then 1:1 gave the product (795 mg, 73%) as micro­crystalline plates, DSC m.p. 145.2 °C. ^1^H NMR δ = 0.79 (3H, d, J = 7.2 Hz, 9-Me), 0.92 (3H, d, J = 6.4 Hz, 6-Me), 0.94-1.01 (1H, m), 1.17-1.23 (2H, m), 1.28-1.34 (2H, m), 1.37 (3H, s, 3-Me), 1.40-1.46 (1H, m), 1.49-1.53 (1H, m), 1.65-1.69 (2H, m), 1.81-1.85 (1H, m), 1.96-1.98 (1H, m), 2.28-2.33 (1H, m), 2.52-2.65 (2H, m, piperazine), 2.93-3.03 (2H, m, piperazine) 3.37-3.44 (2H, m, piper­azine), 3.66-3.75, (2H, m, piperazine), 4.02 (1H, d, J = 10.2 Hz, H-10), 5.25 (1H, s, H-12), 7.03-7.06 (2H, m, ArH), 7.37-7.39 (2H, m, ArH); 13C NMR δ = 13.40, 20.20, 21.55, 24.65, 25.95, 28.37, 34.16, 36.21, 37.30, 45.69, 51.63, 80.20, 90.59, 91.60, 103.93, 115.31, 115.46, 129.32, 129.37, 169.27. IR (film) ν_max_ = 489, 510, 530, 552, 565, 583, 604, 700, 714, 745, 760, 807, 824, 847, 854, 878, 902, 927, 946, 961, 982, 1013, 1021, 1044, 1053, 1101, 1117, 1129, 1158, 1187, 1208, 1223, 1243, 1262, 1279, 1304, 1326, 1377, 1410, 1438, 1458, 1511, 1602, 1622, 2834, 2876, 2929, 2955 cm^-1^. MS *m/z* calcd for C_26_H_36_FN_2_O_5_^+^ 475.2608 [M+H]^+^; found 475.2619. Analysis by HPLC indicates a purity of 99.6%.

**S.1.2 Preparation of substituted DHA-sulfamide derivatives 29 and 30** (Scheme 2)***.***

(2-Pyridyl)piperazine sulfamide DHA **29**: For preparation of *N*-(2-pyridyl)piperazine sulfamide, a mixture of sulfamide (500 mg, 5.02 mmol) and *N*-(2-pyridyl)­piperazine (0.792 mL, 5.02 mmol, 1.0 equiv.) in dimethoxy­ethane (10 mL) under nitrogen was heated at reflux at an oil bath temperature of 110 °C for 24 h. The mixture was then filtered and the filtrate was concentrated by evaporation under reduced pressure to leave a solid residue. This was dissolved in dichloromethane (5 mL), and the solution was washed with water (2 x 5 mL) and brine (2 x 5 mL). The organic layer was separated and dried (MgSO_4_). After filtration to remove the drying agent, the filtrate was evaporated to dryness under reduced pressure to leave the crude *N*-(2-pyridyl)piperazine sulfamide as a white crystalline solid (1.123 g, 89%) that was sufficiently pure to be used in the following step. A sample was recrystallized from dichloro­methane-hexane (1:1) to give the sulfamide as colour­less plates, m.p. 150-151 °C. ^1^H NMR: δ = 3.27-3.30 (m, 4H, piperazine), 3.66-3.69 (m, 4 H, piperazine), 4.50 (s, 2H, NH_2_), 6.67 (2H, d, *J* = 7.9 Hz, pyridyl H), 7.49-7.54 (1H, m, 1H, pyridyl H), 8.20 (1H, d, *J* = 3.8 Hz, pyridyl H). IR (film) ν_max_ 3712, 3691, 3678, 3460, 3445, 2851, 1592, 1560, 1509, 1482, 1459, 1437, 1351, 1329, 1312, 1245, 1160, 1065, 981, 950, 777, 733 cm^-1^; MS (CI, CH_4_) *m/z* (%) 107.0 (4), 133.0 (4), 162.1 (38), 164.1 (100), 242 (14), 243.1 (64). MS (ESI) calcd 243.0916 (M^+^+1), found 243.0941. C_9_H_14_N_4_O_2_S calcd. C 44.61 H 5.82 N 23.11; found C 43.99 H 5.77 N 23.41.

Next, bromotrimethylsilane (0.18 mL, 1.43 mmol, 1.02 equiv.) was added dropwise to a cold (0 °C) stirred solution of 10α-(trimethylsilyloxy)dihydroartemisinin (500 mg, 1.40 mmol) in dichloro­methane (5 mL) under nitrogen at 0 °C (Haynes, 2003; 2004). After 30 min, the solution was transferred via cannula into a stirred solution of the *N*-(2-pyridyl)piperazine sulfamide (680 mg, 2.80 mmol, 2.0 equiv.) in dichloro­methane (15 mL) at 0 °C under nitrogen. After a further 1.5 h, the reaction mixture was treated with saturated aqueous NaHCO_3_ (20 mL). The organic layer was separated, and the aqueous layer was extracted with dichloromethane (3 x 20 mL). The combined organic layer was dried (MgSO_4_). Filtration and concentration of the filtrate under reduced pressure gave a yellow residue, which on chromatography with ethyl acetate-hexane (2:3) gave the product as a white microcrystalline solid (155 mg, 21%), DSC m.p. 155.9 °C. Recrystallization from ethyl acetate-hexane gave fine rods, m.p. 160-161 °C. ^1^H NMR: δ 0.92 (6H, d, J = 5.9 Hz, 6-Me and 9-Me), 0.95-1.02 (1H, m), 1.18 (3H, s, 3-Me), 1.21-1.25 (1H, m), 1.27-1.32 (2H, m), 1.38-1.44 (1H, m), 1.54-1.58 (1H, m), 1.68-1.76 (2H, m), 1.82-1.85 (1H, m), 1.91-1.95 (1H, m), 2.26-2.32 (2H, m), 3.33-3.45 (4H, m, piperazine), 3.66 (4H, m, piperazine), 4.55 (1H, dd, *J* = 10.2 Hz, 10.2 Hz, H-10), 5.03 (1H, d, *J* = 10.1 Hz, NH), 5.23 (1H, s, H-12), 6.61-6.65 (2H, m, pyridyl-H), 7.47 (1H, t, *J* = 7.2 Hz, pyridyl-H), 8.15 (1H, t, *J* = 3.8 Hz, pyridyl-H); ^13^C NMR: δ 13.51, 20.18, 21.60, 24.65, 25.67, 32.76, 33.94, 36.05, 37.33, 45.43, 51.43, 80.13, 80.80, 91.10, 104.22, 113.49. IR (film): ν_max_ = 414, 437, 450, 464, 486, 503, 522, 551, 582, 620, 631, 647, 674, 722, 774, 825, 846, 874, 890, 902, 917, 942, 957, 983, 1024, 1070, 1086, 1095, 1120, 1146, 1198, 1210, 1230, 1250, 1276, 1312, 1339, 1380, 1436, 1481, 1564, 1592, 1728, 2851, 2922, 2945, 3319, 3374 cm^-1^. MS: *m/z* calcd for C_24_H_37_N_4_O_6_S^+^ 509.2434 [M+H]^+^; found 509.2420.

(4-Trifluoromethly-2-pyridyl)piperazine sulfamide DHA **30**: A stirred solution of sulfamide (1.5 g, 15.607 mmol, 1 equiv.) and 1-(2-pyridyl)piperazine 97% (3.72 g, 15.607 mmol, 1 equiv) in 1,2-dimethoxyethane (50 mL) under nitrogen was heated at reflux at an oil bath temperature of 110 °C for 48 h. After that, the solvent was evaporated under reduced pressure. The residue was dissolved in hot ethyl acetate (200 ml) and washed with water (ca. 50 ml X 3), the organic layer was dried with magnesium sulfate, filtered and evaporated under reduced pressure to give a pale yellow solid residue (3.858 g, 80%), that was used without further purification. ^1^H NMR δ = 3.01-3.02 (4H, m, piperazine), 3.73-3.75 (4H, m, piper­azine), 6.85 (2H, s, NH), 7.01 (1H, d, J = 9.01 Hz, pyridyl-H), 7.83 (1H, dd, J = 9.4, 2.7 Hz, pyridyl H), 8.43 (1H, s, pyridyl-H).

Oxalyl chloride (0.35 mL, 1.15 equiv) was slowly added to a stirred mixture of dihydroartemisinin (1.0 g, 3.517 mmol) in toluene (10 mL) containing dimethyl sulfoxide (25 µL, 0.1 equiv) at room temperature under nitrogen. During the addition the dihydroartemisinin dissolved to form a pale amber solution. After 1 h the reaction mixture was transferred via cannula into a stirred solution of *N*-(4-trifluoro­methyl-2-pyridyl)­piperazine sulfamide (1.637 g, 1.5 equiv.) in dichloromethane (15 mL) and the resulting mixture was stirred for 12 h. It was then quenched with saturated aqueous NaHCO_3_ (30 mL) and extracted with ethyl acetate (3 x 25 mL). The extracts were combined, washed with brine (30 mL) and dried over MgSO_4_. After filtration to remove drying agent, the filtrate was evaporated under reduced pressure to leave a solid residue, that after column chromatography on silica gel with ethyl acetate-hexane 1:3 then 3:7 gave the product (387 mg, 19%) as a fine microcrystalline powder, DSC m.p. 149.5 °C. ^1^H NMR δ = 0.91-0.93 (6H, m, 6-Me and 9-Me), 0.95-1.02 (1H, m), 1.18 (3H, s, 3-Me), 1.21-1.25 (1H, m), 1.27-1.31 (2H, m), 1.38-1.44 (1H, m), 1.54-1.58 (1H, m), 1.68-1.76 (2H, m), 1.82-1.86 (1H, m), 1.90-1.95 (1H, m), 2.25-2.30 (2H, m), 3.32-3.43 (4H, m, piperazine), 3.74-3.81 (4H, m, piperazine), 4.53 (1H, dd, J = 10.4 Hz, 10.4 Hz, H-10), 5.01 (1H, d, J = 10.6 Hz, NH), 5.27 (1H, s, H-12), 6.64 (2H, d, J = 9.0 Hz, pyridyl-H), 7.61 (1H, dd, J = 9.1, 1.2 Hz, pyridyl-H), 8.36 (1H, s, pyridyl-H); ^13^C NMR δ 13.47, 20.17, 21.60, 24.63, 25.47, 32.69, 33.92, 35.99, 37.36, 44.55, 45.26, 45.38, 51.40, 80.10, 80.71, 91.06, 104.21. IR (film) ν_max_ = 476, 488, 514, 557, 581, 595, 633, 717, 736, 756, 787, 804, 825, 847, 860, 873, 887, 912, 947, 956, 1016, 1055, 1069, 1080, 1104, 1128, 1149, 1159, 1199, 1254, 1286, 1313, 1329, 1372, 1385, 1430, 1449, 1521, 1566, 1614, 1729, 2855, 2867, 2924, 2949, 2982, 3329 cm^-1^. MS *m/z*: calcd for C_25_H_36_F_3_N_4_O_6_S^+^ 577.2308 [M+H]^+^; found 577.2359.
